# Mice lacking **β**-arrestin-2 in melanocortin 4 receptor–expressing neurons show marked metabolic deficits

**DOI:** 10.1172/jci.insight.202213

**Published:** 2026-04-21

**Authors:** Misbah Rashid, Lei Wang, Zhenzhong Cui, Oksana Gavrilova, Huiyan Lu, Kozo Kaibuchi, Sarah Zeitlmayr, Thomas Gudermann, Andreas Breit, Jürgen Wess

**Affiliations:** 1 Molecular Signaling Section, Laboratory of Bioorganic Chemistry;; 2 Mouse Metabolism Core Laboratory; and; 3 Mouse Transgenic Core Facility, National Institute of Diabetes and Digestive and Kidney Diseases, NIH, Bethesda, Maryland, USA.; 4 Institute for Comprehensive Medical Science, Fujita Health University, Toyoake, Aichi, Japan.; 5 Walther Straub Institute of Pharmacology and Toxicology, Medical Faculty, LMU Munich, Munich, Germany.

**Keywords:** Endocrinology, Metabolism, G protein-coupled receptors, G proteins, Obesity

## Abstract

Hypothalamic melanocortin 4 receptors (MC4Rs) play a central role in regulating food intake and energy homeostasis. In fact, inactivating mutations in the *MC4R* gene are the most common form of monogenic obesity. Agonist activation of MC4Rs reduces food intake by modulating hypothalamic signaling circuits. Thus, a detailed understanding of the signaling pathways that regulate MC4R activity is of considerable translational relevance. Ligand-activated MC4Rs not only interact with heterotrimeric G proteins but also can recruit β-arrestin-2 (barr2) to the receptor. The potential functional role of barr2 in regulating the anorectic effects of MC4R signaling remains unexplored. In the present study, we used mutant mouse models to demonstrate MC4R-mediated activation of barr2/ERK signaling in MC4R neurons of the paraventricular nucleus leads to reduced food intake. We also found the appetite-suppressing effect of setmelanotide, an MC4R agonist FDA approved for the treatment of certain types of obesity, requires the presence of barr2 in MC4R-containing neurons. These data suggest that MC4R agonists able to promote MC4R/barr2 interactions with high efficacy may become useful as appetite-suppressing drugs.

## Abstract 2

We demonstrate that b-arrestin-2 expressed by the paraventricular nucleus of the hypothalamus is critical for melanocortin 4 receptor function.

## Graphical abstract

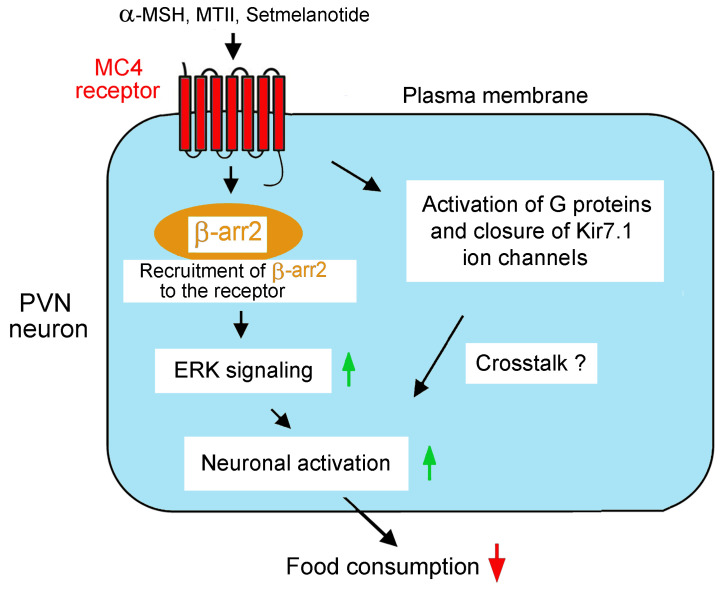

## Introduction

To develop drugs for the prevention and treatment of obesity and associated metabolic disorders, it is essential to identify cellular pathways and signaling molecules that are critical for regulating food intake and energy expenditure. These latter processes are mainly under the control of circuits within the CNS, involving numerous brain regions and signaling pathways (1–3).

It is well established that the central melanocortin system plays a key role in regulating food intake and energy balance in general (4–7). The arcuate nucleus of the hypothalamus (ARC) contains 2 distinct neuronal populations that are key components of the central melanocortin system. One subset of ARC neurons contains and releases agouti-related peptide, which acts as an inverse agonist at melanocortin 4 receptors (MC4Rs) to prevent MC4R signaling, thus promoting food intake and inhibiting energy expenditure. On the other hand, another set of ARC neurons, the so-called pro-opiomelanocortin neurons, secrete α-melanocyte–stimulating hormone, which acts as an agonist at MC4Rs, leading to the suppression of appetite and enhanced energy expenditure (1–3).

The MC4R represents the key signal transducer within the central melanocortin system (4–7). In fact, inactivating mutations in the *MC4R* gene are the most common form of monogenic obesity, characterized by hyperphagia and decreased energy expenditure (4–10). The MC4R is a member of the superfamily of G protein–coupled receptors (GPCRs) and preferentially couples to G_s_, a heterotrimeric G protein that is linked to the activation of adenylyl cyclase and the generation of cAMP (4–8). MC4Rs are expressed primarily in the brain, with particularly high levels present in regions known to be critically involved in the regulation of energy balance, such as the brainstem, nucleus tractus solitarius, thalamus, and hypothalamus (11, 12). In the hypothalamus, the major control center of energy homeostasis, the MC4R is highly enriched in the paraventricular nucleus (PVN) and the dorsomedial hypothalamus (DMH) (13–15).

Given its central role in the regulation of appetite and energy homeostasis, the MC4R is considered a primary therapeutic target for the treatment of obesity (4–8). However, despite decades of research, only 1 MC4R agonist, setmelanotide, has received FDA approval, and its use is currently limited to certain rare forms of genetic obesity, including pro-opiomelanocortin and leptin receptor deficiencies (16–18). A deeper understanding of MC4R function at the molecular level may lead to the development of more efficacious MC4R agonists.

Like other GPCRs, activated MC4Rs recruit a pair of GPCR-regulatory proteins known as β-arrestin-1 and -2 (barr1 and barr2, respectively) (9, 19–21). Beyond their classical roles as inhibitors of GPCR function, β-arrestins also act as signaling proteins in their own right (22, 23). For example, β-arrestins can function as scaffolding proteins capable of modulating various G protein–independent signaling cascades, including the ERK pathway (24, 25). At present, little is known about the potential in vivo roles of β-arrestins in regulating the function of the MC4R or other GPCRs critically involved in the regulation of energy homeostasis.

Lotta et al. (9) recently analyzed the coupling properties of a large number of nonsynonymous missense *MC4R* variants identified in approximately 0.5 million individuals from the UK Biobank. Functional studies with cultured cells transiently expressing these receptor variants revealed that gain-of-function (GoF) *MC4R* variants that displayed preference for barr2 recruitment were linked to a significantly reduced risk of obesity and T2D (9). Interestingly, Lotta et al. (9) observed a positive correlation between the degree of barr2 bias displayed by the different GoF *MC4R* variants and their ability to stimulate agonist-dependent ERK phosphorylation (9).

Based on these findings, we speculated that MC4R-mediated recruitment of barr2 and its downstream signaling pathways might play a key role in mediating the beneficial metabolic effects of MC4R activation. To test this hypothesis, we generated and analyzed mutant mice lacking barr2 specifically in MC4R-expressing cells. By using a combination of different experimental in vivo and in vitro approaches, we demonstrated that MC4R-mediated activation of the barr2/ERK signaling cascade in MC4R-positive neurons of the PVN contributes to the anorectic activity of MC4R agonists.

## Results

### Generation of mutant mice lacking barr2 selectively in MC4R-expressing cells.

To study the role of barr2 in MC4R function, we generated mutant mice that selectively lacked barr2 in MC4R-expressing cells (MC4R-barr2-KO mice). To obtain this mouse strain, we first crossed homozygous floxed barr2 mice (*barr2^fl/fl^*) (26) with MC4R-Cre mice, which express Cre recombinase selectively in MC4R neurons (27). Heterozygous floxed barr2 mice harboring the *MC4R-Cre* transgene were then backcrossed to *barr2^fl/fl^* mice to generate *barr2^fl/fl^ MC4R-Cre* mice (MC4R-barr2-KO mice) and *barr2^fl/fl^* littermates lacking the *MC4R-Cre* transgene. These latter mice served as control littermates throughout all studies (Supplemental Figure 1A; supplemental material available online with this article; https://doi.org/10.1172/jci.insight.202213DS1).

The *barr2^fl/fl^* mice used for these matings had a pure C57BL/6 background (26, 28). The MC4R-Cre mice used by other investigators in the past had a mixed genetic background (27, 29). To avoid potential experimental artifacts associated with the use of mice with a mixed genetic background, we backcrossed the MC4R-Cre mice for at least 5 generations onto the C57BL/6N background C57BL/6NTac.

### Barr2 mRNA expression is greatly reduced in MC4R^+^ neurons.

**Figure 1:**
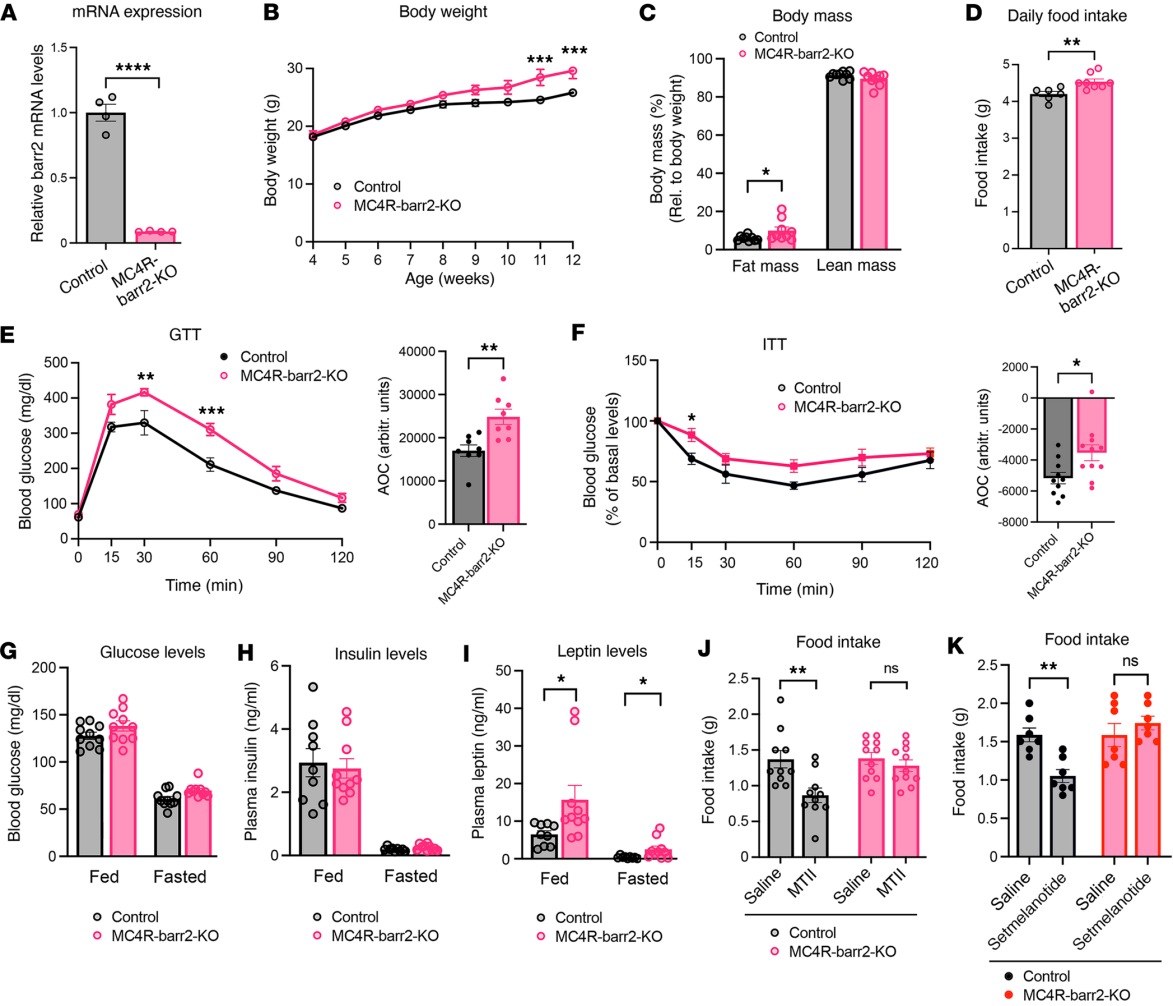
Metabolic analysis of MC4R-barr2-KO mice consuming regular chow. All in vivo experiments were carried out with male mice maintained on regular chow. (**A**) Relative *barr2* mRNA expression levels in hypothalamic MC4R^+^ neurons (mRNA from the PVN and DMH was combined) from the indicated mouse strains (*n* = 4). (**B**) Body weight gain of MC4R-barr2-KO mice and control littermates (control, *n* = 9; MC4R-barr2-KO, *n* = 7). (**C**) Body composition analysis (*n* = 9). (**D**) Daily food intake per mouse was measured for 1 week (*n* = 6–8). (**E**) Glucose tolerance test (GTT). Glucose (2 g/kg) was injected i.p. after an overnight fast (*n* = 8). (**F**) Insulin tolerance test (ITT). After a 4-hour fast, mice were injected with insulin (0.75 U/kg, i.p.) (*n* = 10 or 11). (**G**–**I**) Blood glucose (**G**), plasma insulin (**H**), and plasma leptin (**I**) levels of freely fed and fasted (overnight) mice (*n* = 9 or 10). (**J**) MTII-induced suppression of food intake. After a 24-hour fast, single-housed mice were injected i.p. with either vehicle (saline) or MTII (200 μg) 30 minutes before lights out (6 pm). Food intake was recorded during the first 3.5 hours of the dark phase (*n* = 10 or 11). (**K**) Setmelanotide-induced inhibition of food consumption. After a 24-hour fast, single-housed mice were injected i.p. with either saline or setmelanotide (2 mg/kg) 30 minutes before the start of the dark period. Food intake was measured as described under **J** (*n* = 7; 12–13-week-old mice). Data shown in **C** and **D** were obtained with 12–13-week-old mice. Results shown in **E**–**J** were generated using 14–18-week-old mice. Data are expressed as mean ± SEM. *P < 0.05, **P < 0.01, ***P < 0.001, ****P < 0.0001 (2-way ANOVA followed by Šidák’s multiple-comparison test (**A**–**C**, and **E**–**K**) or 2-tailed Student’s *t* test (**A** and **D**, and AOC bars in **E** and **F**). AOC, area of the curve; PVN, paraventricular nucleus; DMH, dorsomedial hypothalamus.

Since MC4Rs are highly expressed in the PVN and DMH of the hypothalamus (30), we injected an adeno-associated virus (AAV) encoding mCherry into the PVN and DMH of both *MC4R-Cre barr2^fl/fl^* mice (MC4R-barr2-KO mice) and control littermates (MC4R-Cre mice). This virus (AAV8-DIO-mCherry) was constructed in a fashion that directed mCherry expression only in Cre-expressing neurons. By using a FACS strategy, we selectively isolated mCherry-positive (MC4R^+^) neurons and measured *barr2* mRNA expression by qRT-PCR (Supplemental Figure 1B). This analysis showed that *barr2* mRNA levels were greatly reduced in *barr2^fl/fl^ MC4R-Cre* mice (MC4R-barr2-KO mice) compared with their control littermates (Figure 1A). qRT-PCR studies demonstrated that *Mc4r* mRNA levels remained unaffected in the PVN of MC4R-barr2-KO mice (Supplemental Figure 2).

### Insertion of the MC4R-Cre transgene has no significant effects on key metabolic parameters.

To demonstrate that the *MC4R-Cre* transgene insertion itself did not cause unintended metabolic phenotypes, we crossed heterozygous MC4R-Cre mice (genetic background C57BL/6) with WT C57BL/6 mice to generate heterozygous MC4R-Cre mice and WT littermates. These mice were maintained on either regular chow (RC) or a high-fat diet (HFD) and subjected to a series of metabolic tests. We found that the heterozygous MC4R-Cre mice and WT littermates did not differ significantly in body weight, food intake, glucose tolerance, insulin tolerance, or blood glucose levels (Supplemental Figure 3, A–I). Moreover, i.p. injection of Melanotan II (MTII, 200 μg), an MC4R agonist, reduced food intake to a similar degree in WT and MC4R-Cre mice on an HFD (Supplemental Figure 3J). These findings indicated that the presence of the *MC4R-Cre* transgene had no significant effect on the metabolic parameters measured in the present study.

### Metabolic phenotypes of MC4R-barr2-KO mice consuming RC.

We first subjected MC4R-barr2-KO mice and their control littermates consuming RC to a series of metabolic tests. Until 8 weeks of age, the 2 groups of mice did not differ in body weight (Figure 1B). However, starting from week 9, MC4R-barr2-KO mice gained significantly more weight than their control littermates (Figure 1B and Supplemental Figure 4A). This increase in body weight was associated with a significant increase in fat mass and daily food intake (Figure 1, C and D). Lean body mass did not differ between the 2 groups of mice (Figure 1C). Indirect calorimetry studies failed to reveal any significant differences in total energy expenditure (TEE), respiratory exchange ratio (RER), and locomotor activity between the 2 groups of mice, independent of whether the mice were housed at room temperature (22°C) or at thermoneutrality (30°C) (Supplemental Figure 5, A–C). Taken together, these findings suggest that the increases in body weight and adiposity observed with adult MC4R-barr2-KO mice are due to increased food intake.

Additional metabolic tests demonstrated that MC4R-barr2-KO mice showed impaired glucose tolerance (Figure 1E) and reduced insulin tolerance (Figure 1F), with no differences in fed and fasted blood glucose and plasma insulin levels (Figure 1, G and H). Plasma leptin levels were significantly higher in fed and fasted MC4R-barr2-KO mice, as compared with their control littermates (Figure 1I), consistent with the increase in adiposity caused by the barr2 mutation (Figure 1C).

It is well known that agonist activation of neuronal MC4Rs leads to a profound suppression in appetite (4–7, 31). To test the potential ability of barr2 to modulate this effect, we injected MC4R-barr2-KO mice and their control littermates with the MC4R agonist MTII (200 μg, i.p.) or saline 30 minutes prior to the onset of the dark cycle (6 pm). MTII and α-melanocyte–stimulating hormone, the endogenous MC4R agonist, are both short peptides, but MTII has a significantly longer plasma half-life (32, 33). Besides the MC4R, MTII can also activate other melanocortin receptor subtypes including the MC1R and MC3R (34–36). Early studies with MC4R mutant mice demonstrated that the appetite-suppressing effects of MTII are mediated by MC4Rs expressed by PVN neurons (37, 38).

Strikingly, although MTII (200 μg, i.p.) markedly reduced food intake in control mice, it failed to suppress feeding in MC4R-barr2-KO mice (Figure 1J). Food intake was measured during the initial phase of the dark cycle. These findings indicate that the anorectic effect caused by activation of MC4Rs requires the presence of barr2 expressed by MC4R neurons.

Indirect calorimetry studies showed that MTII (10 mg/kg, i.p.) led to comparable increases in TEE and reductions in RER in MC4R-barr2-KO mice and control littermates, indicating that barr2 deficiency selectively affects MC4R-mediated suppression of food intake (Supplemental Figure 6, A and B).

In 2020, setmelanotide, an MC4R agonist (note that this drug can also activate other melanocortin receptor subtypes) (39), was approved by the FDA for the treatment of obesity caused by various genetic conditions (5, 18). Rodríguez Rondón et al. (40) recently showed that setmelanotide treatment of MC4R-expressing cells stimulates both cAMP production and barr2 recruitment with high potency. Thus, we next explored the effect of barr2 deficiency on the ability of setmelanotide to suppress food intake. Specifically, we injected fasted MC4R-barr2-KO mice and their control littermates with setmelanotide (2 mg/kg, i.p.), followed by measuring food intake during the initial phase of the dark cycle. As expected, setmelanotide treatment of control mice led to a significant reduction in food intake (Figure 1K). In contrast, this effect was absent in setmelanotide-treated MC4R-barr2-KO mice (Figure 1K). This observation strongly suggests that setmelanotide stimulation of MC4Rs requires barr2 recruitment for its appetite-suppressing activity.

### Studies with MC4R-barr2-KO mice maintained on an HFD.

**Figure 2:**
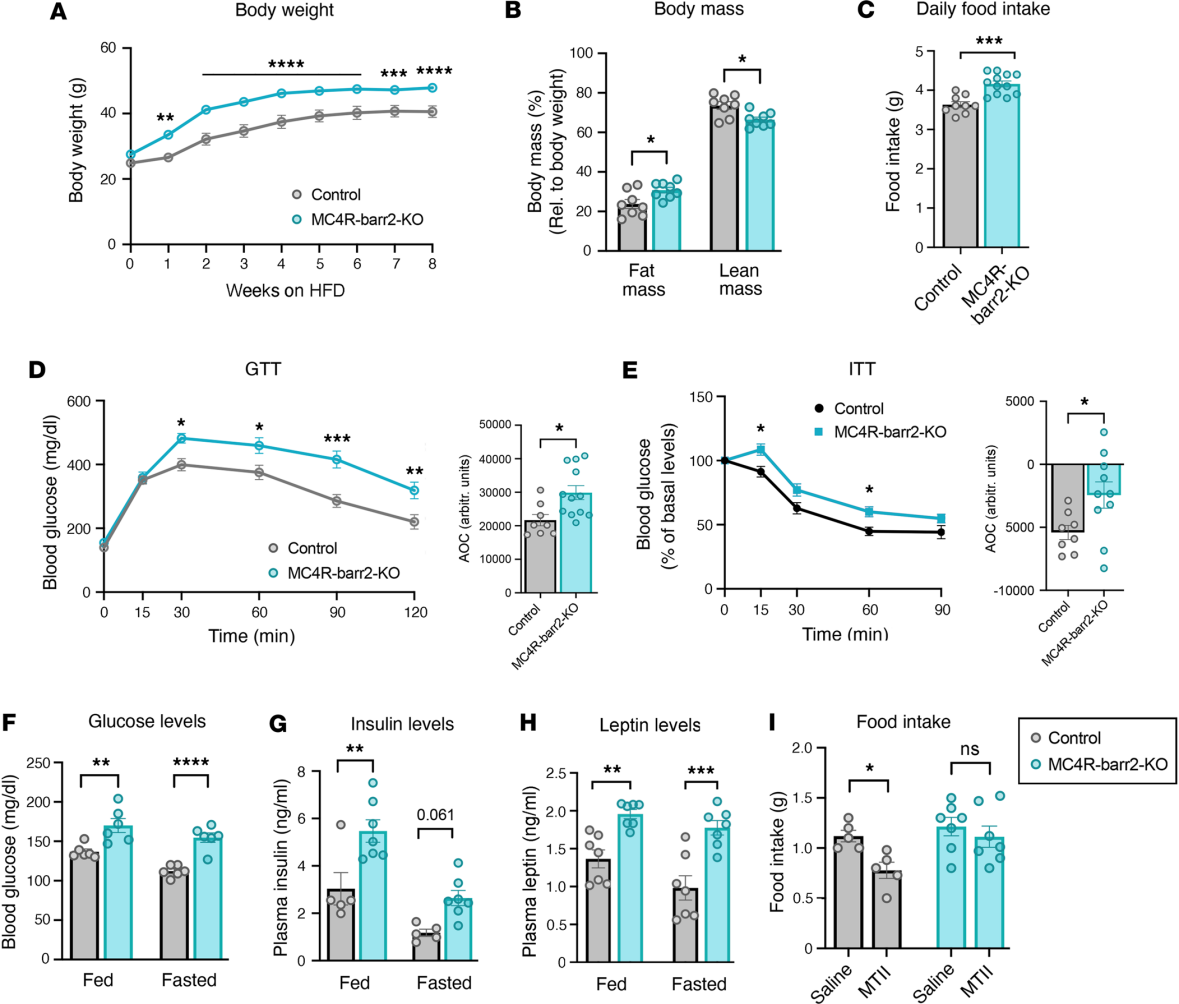
Metabolic studies with MC4R-barr2-KO mice consuming an obesogenic HFD. All experiments were carried out with male mice maintained on an HFD. (**A**) Body weight gain of MC4R-barr2-KO mice and control littermates (*n* = 8). Mice started to consume the HFD when they were 8–9 weeks old. (**B**) Body composition analysis (*n* = 8). (**C**) Food intake studies. Daily food intake per mouse was measured for 1 week (control, *n* = 9; MC4R-barr2-KO, *n* = 12). (**D**) Glucose tolerance test (GTT). After an overnight fast, mice received an i.p. injection of glucose (1 g/kg) (*n* = 8–12). (**E**) Insulin tolerance test (ITT). After a 4-hour fast, mice were injected with insulin (1 U/kg, i.p.) (*n* = 8–11). (**F**–**H**) Blood glucose levels (**F**), plasma insulin levels (**G**), and plasma leptin levels (**H**) of freely fed and fasted (overnight for 16 hours) mice (*n* = 5–7). (**I**) MTII-induced suppression of food intake. After a 24-hour fast, single-housed mice were injected i.p. with either vehicle (saline) or MTII (200 μg) 30 minutes before lights out (6 pm). Food intake was recorded during the first 3.5 hours of the dark phase (*n* = 5–7). Results shown in **B** and **C** were obtained with 12- to 13-week-old mice. Data displayed in **D**–**H** were generated with 14- to 18-week-old mice. Data are expressed as mean ± SEM. *P < 0.05, **P < 0.01, ***P < 0.001, ****P < 0.0001 (2-way ANOVA followed by Šidák’s multiple-comparison test (**A**, **B**, and **D**–**I**) or 2-tailed Student’s *t* test (**C**) and AOC bars in **D** and **E**. AOC, area of the curve.

To assess the potential role of barr2 in MC4R-expressing neurons under obesogenic conditions, MC4R-barr2-KO mice and control littermates were maintained on an HFD (60% kcal from fat) for at least 8 weeks. The mice were 8–9 weeks old when they started consuming the HFD. Under these experimental conditions, MC4R-barr2-KO mice showed a rapid and sustained increase in body weight compared with their control littermates (Figure 2A and Supplemental Figure 4B). This phenotype was considerably more pronounced than the increase in body weight observed with MC4R-barr2-KO mice consuming RC (Figure 1B). MC4R-barr2-KO mice maintained on the HFD also showed a significant increase in the percentage of body fat mass, associated with a significant decrease in the percentage of lean body mass (Figure 2B). Moreover, HFD MC4R-barr2-KO mice showed a significant increase in daily food intake compared with their HFD control littermates (Figure 2C).

Indirect calorimetry studies performed during the second week of HFD exposure did not reveal any reduction in energy expenditure or locomotor activity in the MC4R-barr2-KO mice (Supplemental Figure 7, A and C). At the time of testing, HFD barr2 mutant mice were approximately 6 g heavier than their HFD control littermates (KO, 33.5 ± 0.8; control, 26.6 ± 1.2; *P* < 0.001) and displayed significantly increased TEE (at 22°C and 30°C) and RER (at 30°C) compared with HFD control littermates (Supplemental Figure 7, A and B). Locomotor activity was comparable in the 2 groups of mice (Supplemental Figure 7C). These findings are consistent with previous data showing that elevated mouse body mass is associated with increases in TEE and RER (41). Taken together, our data suggest that the accelerated weight gain observed with HFD MC4R-barr2-KO mice is primarily due to increased food intake.

Additional metabolic studies showed that HFD MC4R-barr2-KO mice also displayed significantly impaired glucose and insulin tolerance (Figure 2, D and E). Moreover, blood glucose and plasma insulin and leptin levels were elevated in both fasted and freely fed HFD MC4R-barr2-KO mice compared with their HFD control littermates (Figure 2, F–H), indicative of impaired insulin sensitivity. Since these metabolic phenotypes are usually observed with obese mice, it is highly likely that the deficits in glucose and insulin tolerance (sensitivity) displayed by the HFD MC4R-barr2-KO mice are a consequence of the increased adiposity caused by the lack of barr2 in MC4R^+^ neurons.

To investigate the effect of barr2 deficiency on the ability of MC4R-mediated suppression of food intake, we injected fasted HFD MC4R-barr2-KO mice and their control littermates with the MC4R agonist MTII (200 μg, i.p.). Consistent with the data obtained with mice consuming RC, MTII treatment failed to inhibit food intake in HFD MC4R-barr2-KO mice (Figure 2I). In contrast, MTII-injected HFD control littermates showed a pronounced decrease in food consumption (Figure 2I). These data indicate that the anorectic effect triggered by MC4R signaling requires the presence of barr2, independent of the type of diet on which the mice are maintained (RC or HFD).

As observed with RC mice, MTII treatment (10 mg/kg, i.p.) affected TEE (significant increase) and RER (significant decrease) in a very similar fashion in HFD-fed MC4R-barr2-KO mice and control littermates (Supplemental Figure 6, C and D), thus providing additional evidence that the lack of barr2 selectively interferes with MC4R-induced inhibition of food intake.

### MC4R-mediated activation of ERK signaling requires the presence of barr2.

To investigate the mechanisms through which barr2 regulates MC4R function, we carried out a series of in vitro studies using hypothalamic mHypoA-2/10 cells. These cells were immortalized from primary hypothalamic cultures of adult male C57BL/6 mice (42). Most importantly, mHypoA-2/10 cells express endogenous MC4Rs (43). For these reasons, mHypoA-2/10 cells are considered a useful in vitro model system for studying the function of neuronal MC4Rs.

**Figure 3:**
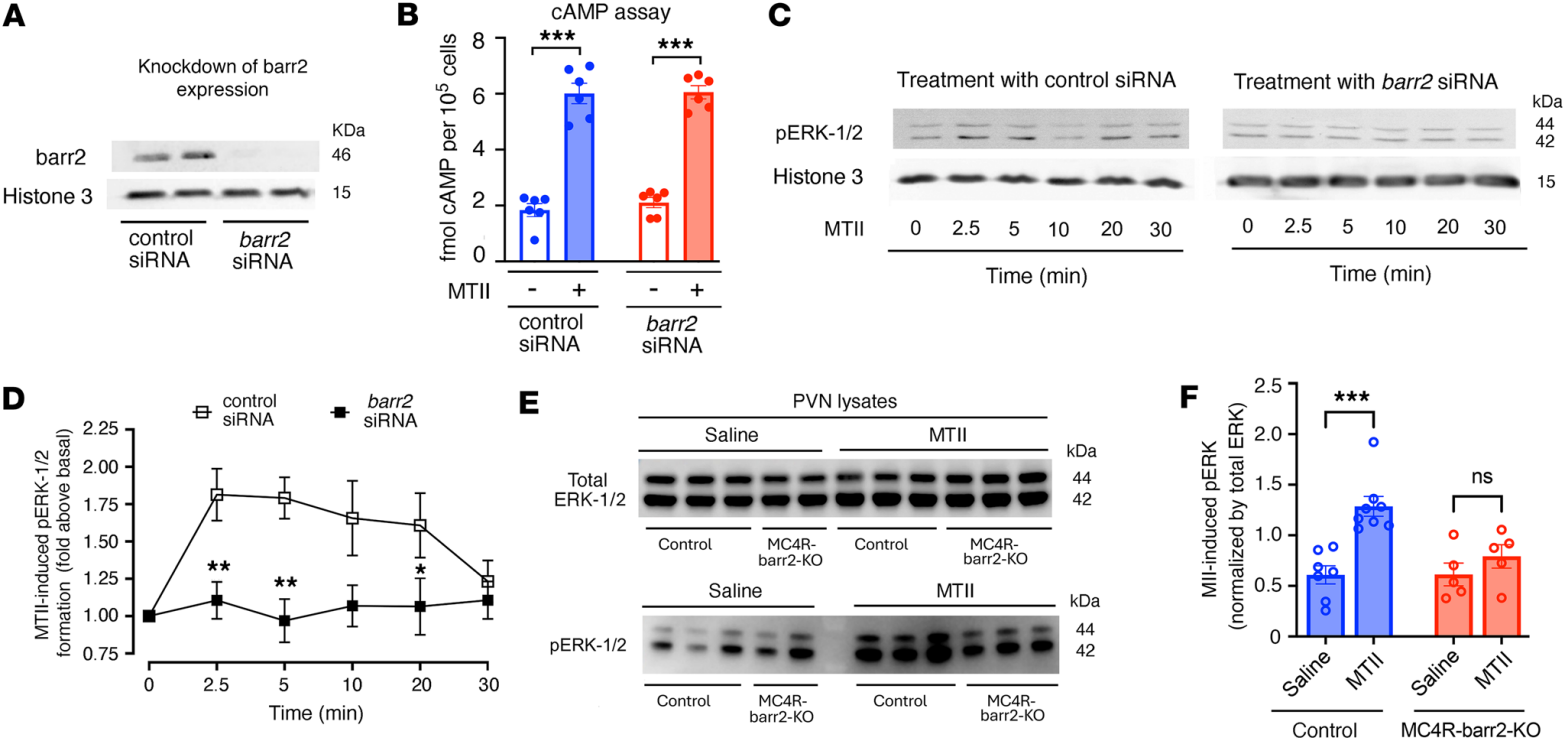
Barr2 deficiency prevents MTII stimulation of pERK-1/2 formation. (**A**–**D**) Studies with mHypoA-2/10 cells. (**A**) Western blot indicating that treatment of mHypoA-2/10 cells with *barr2* siRNA greatly reduces the expression of barr2 protein. Histone 3 served as a loading control. (**B**) MTII-stimulated cAMP accumulation is not affected by knockdown of barr2 expression. HypoA-2/10 cells were incubated in the presence or absence of MTII (100 nM) for 45 minutes. Cellular cAMP levels were determined by sequential chromatography and expressed as fmol cAMP per 10^5^ cells (*n* = 6). (**C**) MTII stimulation of pERK-1/2 formation in mHypoA-2/10 cells. The 2 panels display representative Western blots showing time-dependent effects of MTII (100 nM) on pERK-1/2 levels. Cells were treated with either scrambled control siRNA (left panel) or *barr2* siRNA (right panel). (**D**) Summary of Western blotting data carried out with HypoA-2/10 cells (MTII-induced pERK formation) (*n* = 7). Data are presented as fold-change in pERK-1/2 formation over basal normalized to histone 3 expression. (**E**) MTII stimulation of pERK-1/2 formation in the PVN. MC4R-barr2-KO mice and control littermates (age 15–16 weeks) were injected i.p. with either saline or MTII (10 mg/kg). Thirty minutes later, mice were euthanized, and PVN lysates were prepared and subjected to immunoblotting studies using antibodies directed against pERK-1/2 and total ERK-1/2. Representative Western blots are shown. Each lane represents PVN lysate from a single mouse. Blots for pERK and total ERK were set up in parallel and run contemporaneously. (**F**) Summary of Western blotting data carried out with PVN lysates (MTII-induced pERK formation). Data are presented as fold-change in pERK formation relative to the expression of total ERK (control, *n* = 7; MC4R-barr2-KO, *n* = 5). Data are expressed as mean ± SEM. *P < 0.05, **P < 0.01, ***P < 0.001 (2-way ANOVA with Šidák’s post hoc test). PVN, paraventricular nucleus.

Since the MC4R preferentially couples to G_s_, we first tested whether barr2 deficiency affected MTII-stimulated cAMP accumulation. As shown in Figure 3A, incubation of mHypoA-2/10 cells with *barr2* siRNA led to a robust knockdown of barr2 protein expression. cAMP assays showed that MTII treatment (100 nM) of mHypoA-2/10 cells resulted in comparable increases in intracellular cAMP levels in cells treated with scrambled control siRNA and cells treated with *barr2* siRNA (Figure 3B).

Activated MC4Rs can also couple to G proteins of the G_q_ family, resulting in elevated cytoplasmic Ca^2+^ levels (44, 45). For this reason, we also monitored free intracellular Ca^2+^ concentrations ([Ca^2+^]_i_) by using a Fura-2-AM–based imaging technique (see Methods for details). Treatment of mHypoA-2/10 cells with MTII (1 μM) did not result in any detectable changes in [Ca^2+^]_i_ (Supplemental Figure 8). In contrast, bradykinin (100 nM), an agonist at G_q_-coupled bradykinin receptors, caused a robust increase in [Ca^2+^]_i_ (Supplemental Figure 8), indicating that activation of MC4Rs endogenously expressed by mHypoA-2/10 cells does not lead to the stimulation of G_q_-type G proteins.

As is the case with most other GPCRs, activated MC4Rs can interact with β-arrestins (9, 19–21). Numerous studies have shown that GPCR recruitment of β-arrestins can lead to the activation of MAP kinases (e.g., ERK) under different experimental conditions (22, 23). Interestingly, a recent study (9) reported that human GoF *MC4R* mutations that are biased for barr2 recruitment also led to increased ERK signaling, and that both of these effects are associated with a lean phenotype. Based on these observations, we investigated whether barr2 deficiency in mHypoA-2/10 cells affected MTII-stimulated ERK signaling. As expected, MTII (100 nM) treatment of mHypoA-2/10 cells led to a robust increase in ERK-1/2 phosphorylation in control cells (Figure 3, C and D). Strikingly, this response was virtually abolished after barr2 knockdown with *barr2* siRNA (Figure 3, C and D), indicating that barr2 recruitment is essential for MC4R-mediated ERK activation in MC4R-expressing neurons.

To study MC4R-stimulated ERK signaling ex vivo, we carried out immunoblotting studies using PVN lysates prepared from MC4R-barr2-KO mice and control littermates. Both groups of mice were injected i.p. with either MTII (10 mg/kg) or saline. Thirty minutes later, PVN tissues were collected via micro-punch, lysed, and subjected to Western blotting studies. Consistent with the in vitro data, MTII strongly enhanced pERK-1/2 formation in the PVN of control mice (Figure 3, E and F). In contrast, this response was absent in the PVN of MC4R-barr2-KO mice (Figure 3, E and F). Taken together, these results clearly indicate that the presence of barr2 is critical for MC4R-stimulated ERK signaling.

### Inhibition of ERK signaling in PVN MC4R neurons recapitulates the MC4R-barr2-KO phenotype.

**Figure 4:**
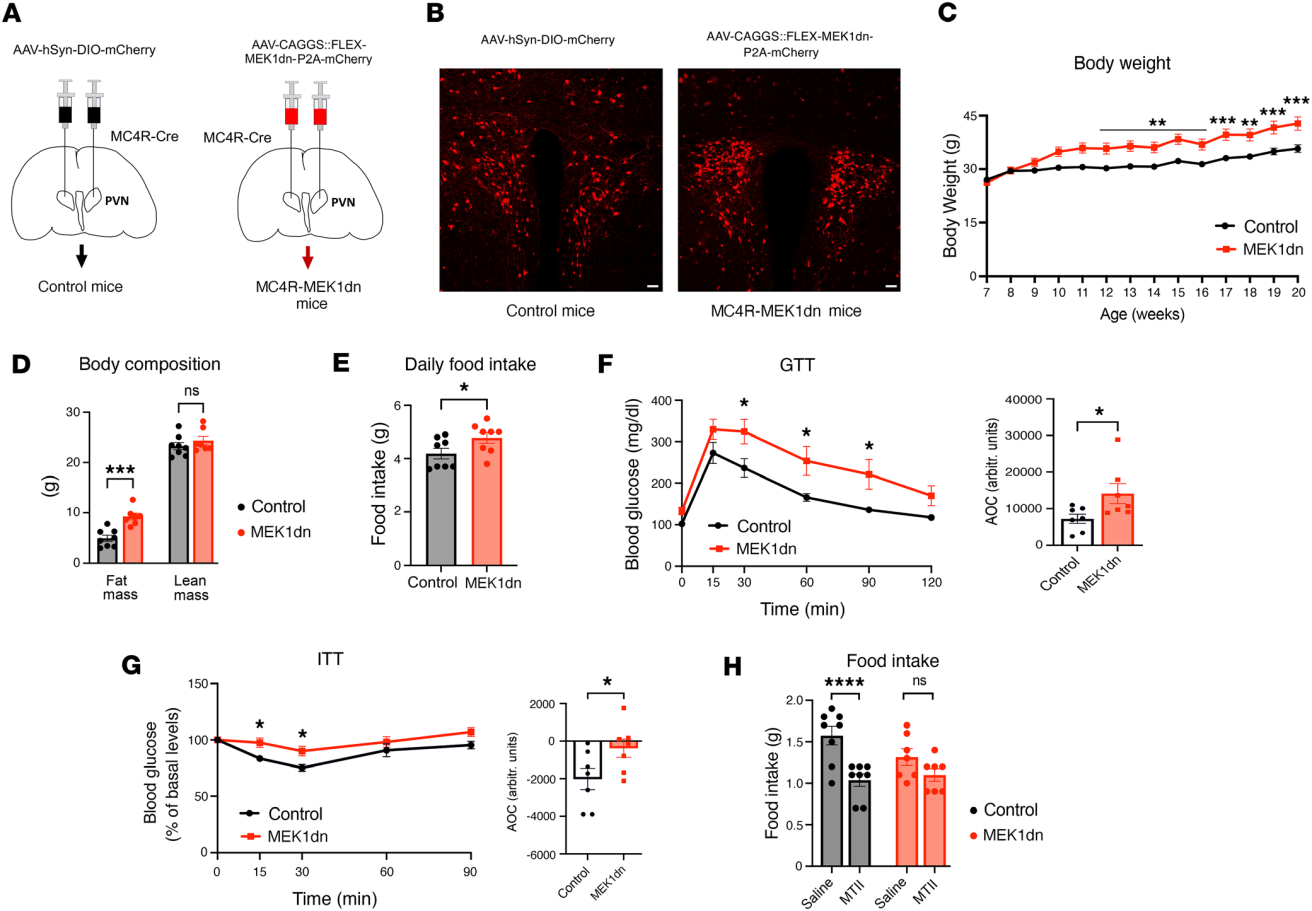
Expression of a dominant-negative mutant of MEK1 in the PVN of MC4R-Cre mice mimics the metabolic phenotypes of MC4R-barr2-KO mice. All experiments were performed using male MC4R-Cre mice consuming regular chow. (**A**) Scheme illustrating the generation of experimental animals: 7-week-old MC4R-Cre mice were bilaterally injected into the PVN with either the AAV-DIO-mCherry control virus (control mice) or the AAV-CAGGS:FLEX-MEK1dn-P2A-mCherry virus leading to the expression of a dominant-negative (dn) MEK1 mutant in MC4R-expressing neurons of the PVN (MC4R-Mekdn mice). (**B**) Representative images showing that the PVN was properly targeted by the 2 viruses (note that the MEK1dn virus contained an mCherry reporter sequence). Scale bar: 100 μm. (**C** and **D**) Body weight gain (**C**) and body composition (**D**) of mice of the indicated genotypes (mouse age in **D** was 16 weeks) (*n* = 7 or 8). (**E**) Daily food intake per mouse measured for 1 week (mouse age 17–18 weeks; *n* = 8). (**F**) Glucose tolerance test (GTT). After an overnight fast, mice received an i.p. injection of glucose (2 g/kg; mouse age 14–15 weeks) (*n* = 7). (**G**) Insulin tolerance test (ITT). After a 4-hour fast, mice were injected i.p. with insulin (0.75 U/kg; mouse age 14–15 weeks) (*n* = 7). (**H**) MTII-induced inhibition of food intake. After a 24-hour fast, single-housed mice were injected i.p. with either vehicle (saline) or MTII (200 μg) 30 minutes before lights out (6 pm). Food intake was recorded during the first 3.5 hours of the dark phase (mouse age: 12–14 weeks) (*n* = 7 or 8). *P < 0.05, **P < 0.01, ***P < 0.001, ****P < 0.0001 (2-way ANOVA with Šidák’s multiple-comparison test (**C**, **D**, and **F**–**H**) or 2-tailed Student’s *t* test (**E**, AOC bars in **F** and **G**). dn, dominant negative; AOC, area of the curve.

Given the observation that barr2 mediates MC4R-activated ERK signaling in MC4R neurons, we tested the hypothesis that impaired ERK signaling is responsible for the inability of activated MC4Rs to suppress food intake in MC4R-barr2-KO mice. As mentioned earlier, MC4Rs expressed by PVN neurons are primarily involved in MC4R regulation of food consumption. Based on these findings, we selectively expressed a dominant-negative mutant of MEK1 (MEK1dn) in PVN MC4R neurons of MC4R-Cre mice to selectively inhibit ERK signaling (Figure 4A). Selective expression of the MEK1dn protein in PVN-MC4R neurons was achieved via bilateral microinjection of the AAV8:FLEX-MEK1dn-P2A-mCherry virus (46) into the PVN of MC4R-Cre mice (note that MEK1dn expression is Cre-dependent; Figure 4B).

**Figure 5:**
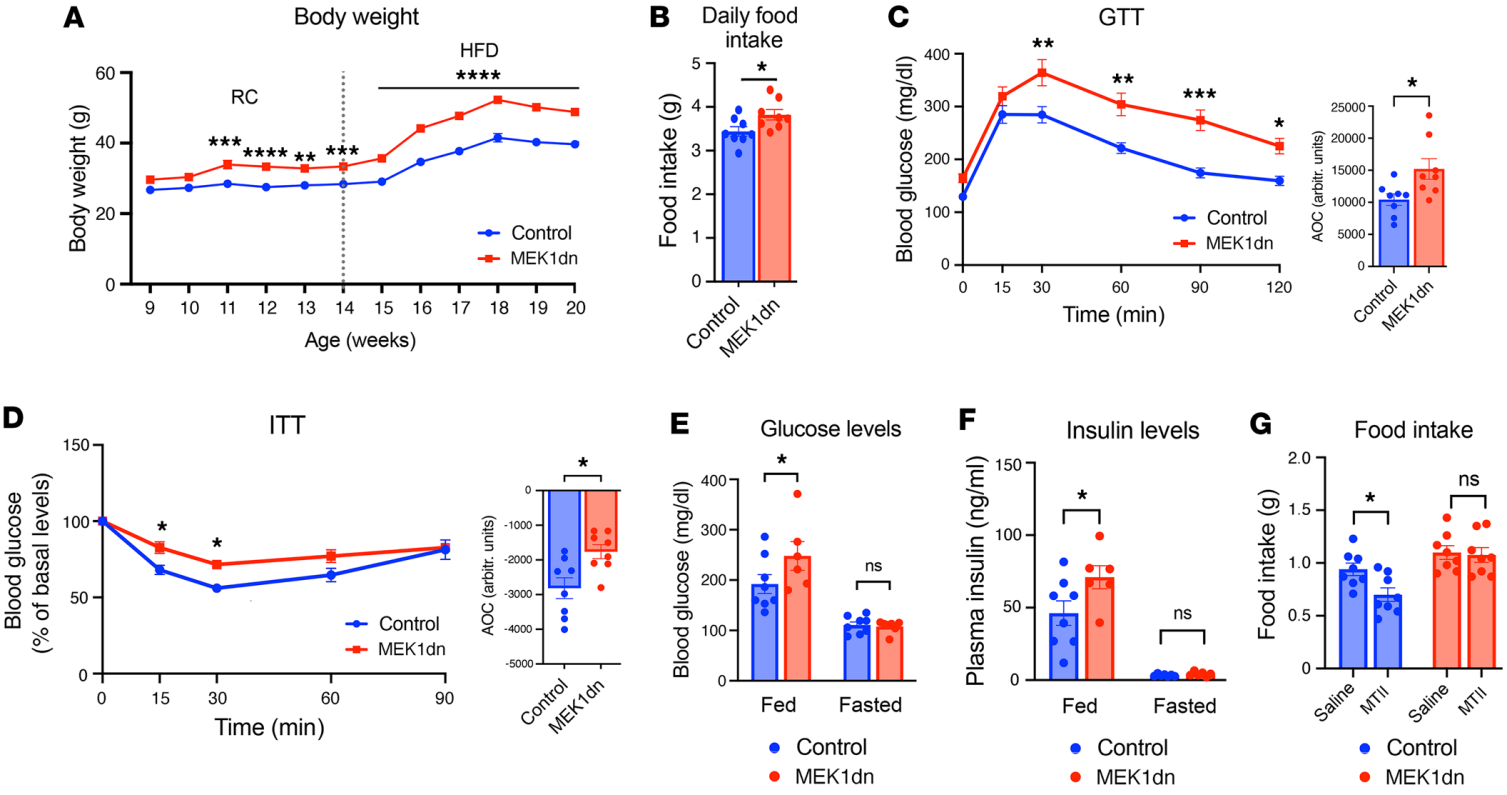
Metabolic phenotypes of MC4R-Mek1dn mice maintained on an HFD. All experiments were carried out with male MC4R-Mek1dn mice. These mice express a dominant-negative mutant of MEK1 in MC4R neurons of the PVN. MC4R-Cre mice expressing mCherry in MC4R neurons of the PVN served as control animals (see Figure 4A for details). (**A**) Body weight gain of mice switched from regular chow (RC) to an HFD (*n* = 7 or 8). (**B**) Daily food intake per day of single-housed mice monitored over a 3-week period (mouse age 14–17 weeks) (*n* = 8). (**C**) Glucose tolerance test (GTT). After an overnight fast, mice received an i.p. glucose bolus (1 g/kg; mouse age 19–20 weeks) (*n* = 8). (**D**) Insulin tolerance test (ITT). After a 4-hour fast, mice were injected i.p. with insulin (1 U/kg) (mouse age 18–19 weeks; *n* = 7 or 8). (**E** and **F**) Blood glucose (**E**) and plasma insulin levels (**F**) under fed and fasted conditions (control, *n* = 8; MC4R-Mek1dn, *n* = 6) (mouse age 20–21 weeks). (**G**) MTII-induced inhibition of food intake. After a 24-hour fast, single-housed mice were injected i.p. with either saline or MTII (200 μg) 30 minutes before lights out (6 pm). Food intake was recorded during the first 3.5 hours of the dark phase (mouse age 17–18 weeks, *n* = 8). Data are presented as mean ± SEM. Statistical significance was assessed using *P < 0.05, **P < 0.01, ***P < 0.001, ****P < 0.0001 (2-way ANOVA followed by Šidák’s multiple-comparison test in **A** and **C**–**G** or unpaired 2-tailed Student’s *t* test **B** and bars in **C** and **D**. dn, dominant negative; AOC, area of the curve.

Intriguingly, mice expressing MEK1dn in PVN MC4R neurons (MC4R-MEK1dn mice) displayed similar metabolic deficits as MC4R-barr2-KO mice (Figures 4 and 5). The MEK1dn mutant mice showed significant increases in body weight, fat mass, and daily food intake, accompanied by impaired glucose and insulin tolerance. These phenotypes were observed with mice maintained on an RC diet (Figure 4, C–G, and Supplemental Figure 4C) or an HFD (Figure 5, A–F). Importantly, MTII was unable to suppress food intake in PVN MC4R-MEK1dn mice. Again, this effect was observed independent of the diet that the mice consumed (RC or HFD; Figure 4H and Figure 5G, respectively).

### MC4R agonist stimulation of pERK formation in PVN MC4R^+^ neurons in vivo is barr2 dependent.

**Figure 6:**
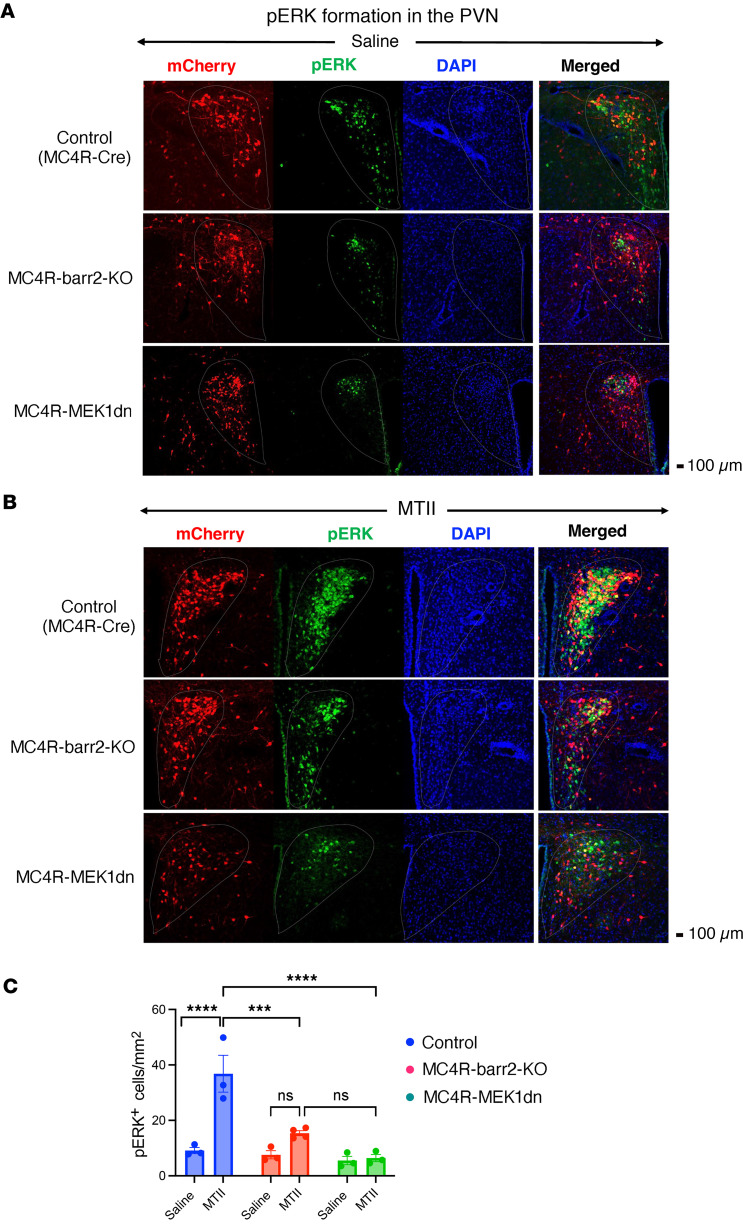
MTII-stimulated pERK formation in MC4R-expressing neurons of the PVN. The expression of pERK in MC4R-expressing neurons of the PVN was studied via immunostaining using hypothalamic slices prepared from *MC4R-*Cre (control), MC4R-barr2-KO, and MC4R-Mek1dn mice. Mice were bilaterally injected into the PVN with AAV-DIO-mCherry (*MC4R*-Cre and MC4R-barr2-KO mice) or AAV8:FLEX-MEK1dn-P2A-mCherry (MC4R-Mek1dn mice) to label MC4R-expressing neurons in the PVN. After a 3-hour fast, animals received an i.p. injection of either vehicle (saline) or MTII (10 mg/kg). Brains were collected 1 hour later. The PVN region is outlined with stippled lines. (**A** and **B**) Immunostaining for pERK in MC4R-expressing neurons in the PVN of mice injected with either vehicle (**A**) or MTII (**B**). Scale bar: 100 μm. (**C**) Quantification of ERK expression data. pERK^+^ cells represent neurons coexpressing mCherry, a marker for MC4R^+^ neurons. Six hypothalamic slices prepared from 3 or 4 different mice per strain were analyzed. Data are presented as mean ± SEM. ***P < 0.001, ****P < 0.0001 (2-way ANOVA followed by Šidák’s multiple-comparison test). PVN, paraventricular nucleus; ns, no statistically significant difference.

To demonstrate that activation of PVN MC4Rs stimulated ERK signaling in vivo, we injected MC4R-barr2-KO mice and control littermates (MC4R-Cre mice) bilaterally into the PVN with the AAV8-DIO-mCherry virus to label MC4R-expressing neurons. Mice were fasted for 3 hours and then injected i.p. with either vehicle (saline) or MTII (10 mg/kg), and brain tissue was collected 60 minutes later. Hypothalamic sections were prepared and subjected to immunofluorescence studies to detect the formation of pERK in PVN MC4R^+^ neurons. We found that MTII treatment of control mice led to a robust increase in the number of pERK-positive PVN MC4R^+^ neurons (Figure 6, A–C). Strikingly, the number of pERK-positive PVN MC4R^+^ neurons was greatly reduced in MC4R-barr2-KO mice (Figure 6, A–C, and Supplemental Figure 9).

We carried out similar experiments with MC4R-Cre mice that had been injected into the PVN (bilaterally) with the AAV8-DIO-mCherry virus to label MC4R-expressing neurons and the AAV8:FLEX-MEK1dn-P2A-mCherry virus to prevent MC4R-stimulated pERK formation. As expected, immunofluorescence studies showed that MTII treatment (10 mg/kg, i.p.) of mice expressing MEK1dn in PVN MC4R^+^ neurons failed to stimulate the formation of pERK in these neurons (Figure 6, A–C, and Supplemental Figure 9).

### Agonist stimulation of cFos formation in PVN MC4R^+^ neurons in vivo requires the presence of barr2.

**Figure 7:**
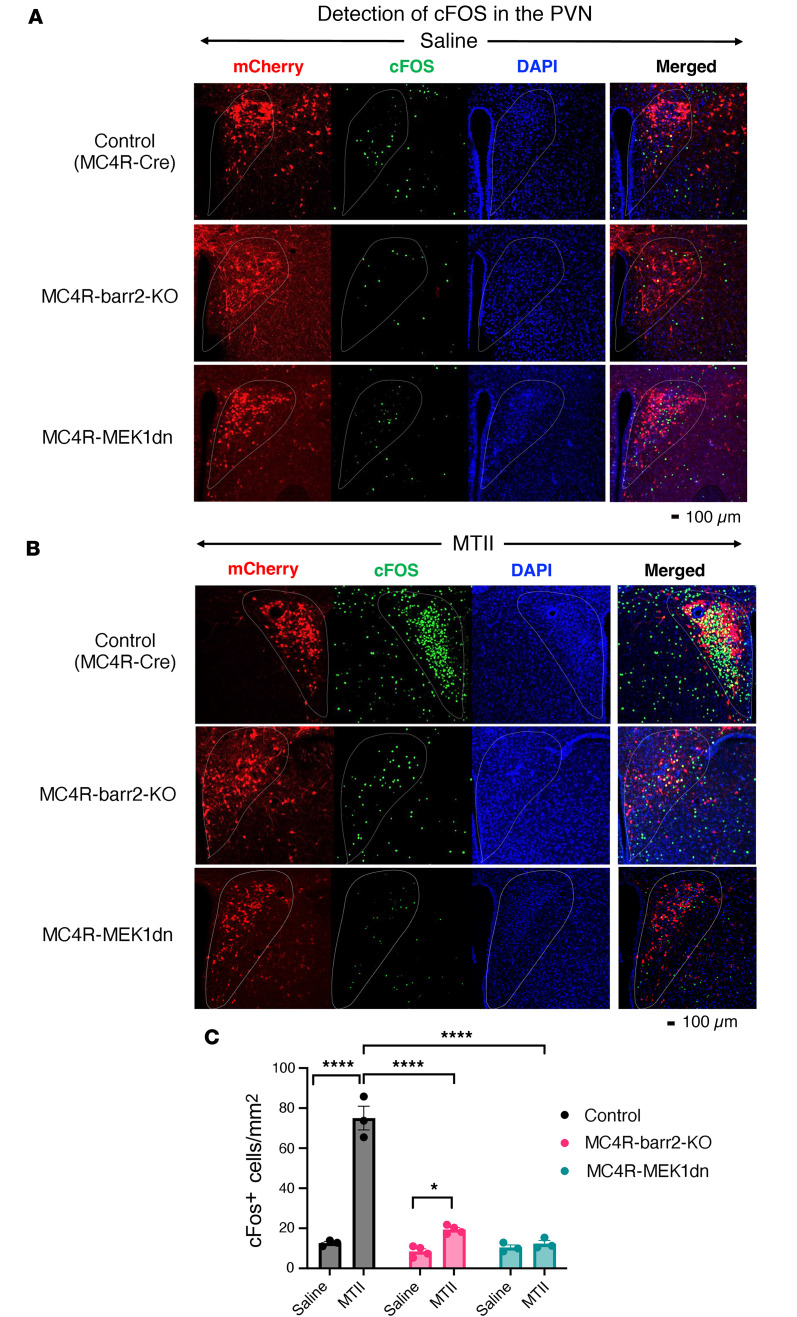
MTII-stimulated cFos expression in MC4R-expressing neurons of the PVN. The expression of cFos in MC4R-expressing neurons of the PVN was studied via immunostaining using hypothalamic slices prepared from *MC4R-*Cre (control), MC4R-barr2-KO, and MC4R-Mek1dn mice. Mice were bilaterally injected into the PVN with AAV-DIO-mCherry (*MC4R-*Cre and MC4R-barr2-KO mice) or AAV8:FLEX-MEK1dn-P2A-mCherry (MC4R-Mek1dn mice) to label MC4R-expressing neurons. After a 3-hour fast, animals received an i.p. injection of either vehicle (saline) or MTII (10 mg/kg). Brains were collected 1 hour later. The PVN region is outlined with stippled lines. (**A** and **B**) Immunostaining for cFos in MC4R-expressing neurons of the PVN of mice injected with either vehicle (**A**) or MTII (**B**). Scale bar: 100 μm. (**C**) Quantification of cFos expression data. cFos^+^ cells represent neurons coexpressing mCherry, a marker for MC4R^+^ neurons. Six hypothalamic slices prepared from 3 or 4 different mice per strain were analyzed. Data are presented as mean ± SEM. *P < 0.05, ****P < 0.0001 (2-way ANOVA followed by Šidák’s multiple-comparison test). PVN, paraventricular nucleus.

We performed additional experiments to explore whether MTII stimulation of PVN MC4R^+^ neurons was able to promote the formation of cFos, a marker of neuronal activation. Specifically, after a 3-hour fast, we injected MC4R-barr2-KO mice, control littermates (MC4R-Cre mice), and PVN MC4R-MEK1dn mice that expressed mCherry in MC4R neurons with either saline or MTII (10 mg/kg, i.p.). We then harvested brain tissues 60 minutes later and prepared hypothalamic sections for immunostaining studies. MTII treatment of control mice resulted in a strong cFos signal in PVN MC4R+ neurons (Figure 7, A–C; colocalization of pERK and cFos is shown in Supplemental Figure 9). Strikingly, the magnitude of this effect was greatly reduced in PVN MC4R^+^ neurons lacking barr2 (MC4R-barr2-KO mice) (Figure 7, A–C, and Supplemental Figure 9). Similarly, MTII treatment of MC4R-Mek1dn mice failed to increase the number of cFos^+^ cells in PVN MC4R^+^ neurons (Figure 7, A–C, and Supplemental Figure 9). Taken together, these data support the concept that MC4R agonist activation of PVN MC4R^+^ neurons requires MC4R-mediated stimulation of the barr2/ERK signaling cascade.

### Reintroduction of barr2 into PVN MC4R^+^ neurons.

To further strengthen the concept that barr2 expressed by PVN MC4R^+^ neurons represents an important regulator of MC4R-mediated metabolic effects, we reintroduced barr2 into PVN MC4R^+^ neurons of MC4R-barr2-KO mice maintained on an HFD. Specifically, we injected a Cre-dependent AAV (AAV-EF1α-DIO-barr2-myc-P2A-Cerulean) coding for barr2 into the PVN of control mice that did not harbor the *MC4R-Cre* insert (genotype *barr2^fl/fl^*) and into the PVN of MC4R-barr2-KO mice (genotype *barr2^fl/fl^ MC4R-Cre*) (Supplemental Figure 10, A and E). This injection protocol resulted in the AAV-directed expression of barr2 in the PVN of MC4R-barr2-KO mice but not in control mice (Supplemental Figure 10I). For control purposes, we also injected a Cre-dependent AAV (AAV-DIO-EGFP) coding for EGFP, which is pharmacologically inert, into the PVN of control and MC4R-barr2-KO mice (Supplemental Figure 10, A, E, and I).

As expected, MC4R-barr2-KO mice injected with the AAV-DIO-EGFP virus showed similar increases in daily food intake and impairments in glucose homeostasis as untreated MC4R-barr2-KO mice (Supplemental Figure 10, B–D). Strikingly, these deficits were absent after reexpression of barr2 in MC4R^+^ neurons of the PVN of MC4R-barr2-KO mice (Supplemental Figure 10, F–H). In addition, MC4R-barr2-KO mice treated with AAV-EF1α-DIO-Barr2-myc-P2A-Cerulean (bilateral injections into the PVN) displayed a normal anorectic response to treatment with MTII (200 μg per mouse) (Supplemental Figure 11). In sum, these findings corroborate the concept that barr2 expressed by PVN MC4R^+^ neurons plays an important role in mediating the beneficial metabolic effects of MC4R stimulation.

## Discussion

We found that mice lacking barr2 in MC4R-expressing cells (MC4R-barr2-KO mice) displayed a significant increase in adiposity and body weight, enhanced food intake, reduced glucose tolerance, and impaired insulin sensitivity compared with their control littermates. These phenotypes were observed independently of the diet that the mice consumed (RC or HFD; Figures 1 and 2, respectively). Importantly, the ability of systemically administered MC4R agonists to suppress food intake was lost in MC4R-barr2-KO mice (Figure 1, J and K, and Figure 2I). In sum, these data suggest that the obesity phenotype displayed by the barr2 mutant mice was caused by increased food intake, and that the presence of barr2 is required for the anorectic effect of MC4R stimulation.

Since MC4R activation leads to the recruitment of barr2 (9, 21), and barr2 is known to promote ERK-1/2 activation in many cell types (22, 23), we speculated that impaired barr2-mediated ERK-1/2 activation may be responsible for the metabolic deficits displayed by the MC4R-barr2-KO mice. MC4Rs contained within the PVN of the hypothalamus are known to be of primary importance for the appetite-suppressing effects of MC4R activation (27, 38, 47). This concept is strongly supported by the finding that PVN-specific reexpression of MC4Rs in whole-body MC4R-KO mice reversed the hyperphagia displayed by the KO mice but did not affect deficits in energy expenditure (38).

We found that systemic treatment of control mice with MTII strongly promoted the expression of pERK in MC4R^+^ neurons of the PVN (Figure 6, A–C). Strikingly, this effect was absent in PVN MC4R^+^ neurons of MC4R-barr2-KO mice (Figure 6, A–C), suggesting that barr2 recruitment by activated MC4Rs leads to ERK signaling in PVN MC4R^+^ neurons. We obtained similar results with cultured mHypoA-2/10 cells (Figure 3, C and D).

**Figure 8:**
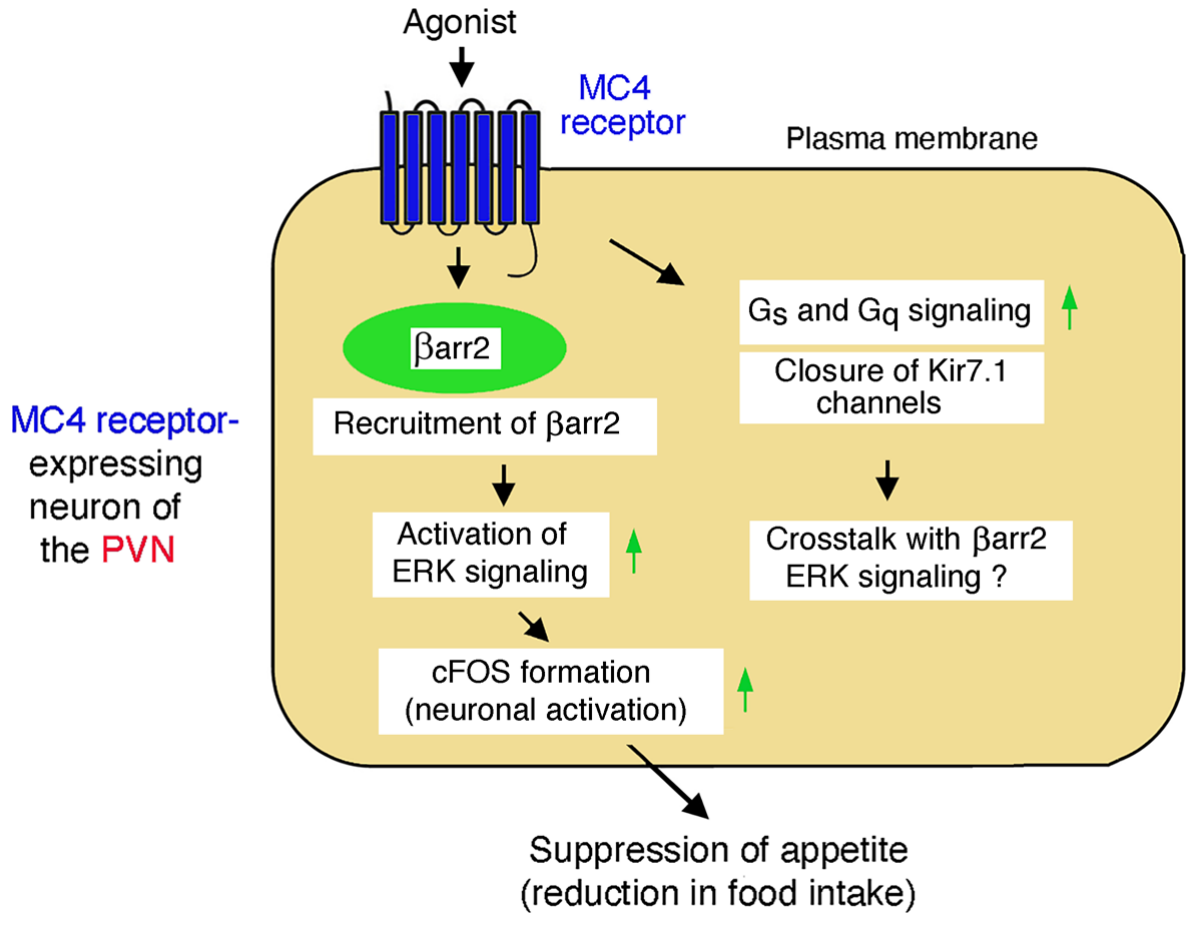
Illustration outlining the role of barr2 in MC4R neurons of the PVN. Activated MC4Rs also stimulate heterotrimeric G proteins (G_s_, G_q_) and cause the closure of Kir7.1 ion channels. It remains to be determined to what extent these pathways modulate the phenotypes displayed by MC4R-barr2-KO mice.

The activation of ERK signaling can lead to neuronal activation through different molecular mechanisms, including the phosphorylation of specific ion channels (48, 49). In the present study, we monitored the expression of cFos as a marker of neuronal activity (50). We found that MTII treatment of control mice strongly promoted the expression of cFos in PVN MC4R^+^ neurons (Figure 7, A–C). On the other hand, this effect was greatly diminished after MTII treatment of MC4R-barr2-KO mice (Figure 7, A–C). These observations strongly suggest that activation of PVN MC4Rs leads to the activation of the barr2/ERK signaling cascade, resulting in neuronal activation and, ultimately, a reduction in food intake (Figure 8).

To further strengthen this conclusion, we carried out additional studies in which we reintroduced barr2 into PVN MC4R^+^ neurons of HFD MC4R-barr2-KO mice. The resulting mutant mice showed reduced food intake and control-like glucose homeostasis and MTII-mediated suppression of food intake (Supplemental Figures 10 and 11).

To provide additional evidence for the existence of this anorectic pathway, we used an AAV-based strategy to express MEK1dn (46), which prevents ERK activation, specifically in MC4R^+^ neurons of the PVN. Strikingly, the resulting mutant mice showed metabolic deficits that closely mimicked those displayed by MC4R-barr2-KO mice. These phenotypes included increases in adiposity, body weight, and food intake (Figure 4, C–G). Importantly, MTII treatment of mice expressing MEK1dn in PVN MC4R^+^ neurons failed to suppress food intake (Figure 4H). In sum, these data strongly support the existence of the barr2-dependent anorectic pathway discussed in the previous paragraph.

Compared with control mice, MC4R-barr2-KO mice exhibited increased adiposity, independent of the diet that the mice consumed. Since elevated body fat mass promotes insulin resistance, it is possible that the increase in adiposity displayed by the MC4R-barr2-KO mice contributes to the observed impairments in glucose tolerance and insulin sensitivity. However, we cannot exclude the possibility that adiposity-independent signaling pathways contribute to the metabolic deficits displayed by the barr2 mutant mice.

Huszar et al. (51) showed that whole-body MC4R-KO mice (males) maintained on RC already weigh 40 g at 7 weeks of age (early-onset obesity). In contrast, at the same age, the weight gain displayed by MC4R-barr2-KO mice (males) consuming RC was delayed and clearly less pronounced (Figure 1B). This observation suggests that MC4R-mediated stimulation of PVN barr2/ERK signaling represents just one pathway contributing to MC4R-mediated weight loss. We would also like to note that the absence of barr2 in MC4R^+^ neurons is likely to interfere with additional cellular functions, besides ERK signaling. For example, it is possible that the lack of barr2 alters the internalization, resensitization, and/or trafficking of the MC4R, and, quite likely, other GPCRs.

The MC4R is broadly distributed across the brain, and each brain region has distinct roles in MC4R-dependent feeding and reward functions (4–7). For this reason, other areas of the brain, besides the PVN, are predicted to contribute to the metabolic phenotypes displayed by the MC4R barr2-KO mice.

Several years ago, Lotta et al. (9) functionally characterized dozens of nonsynonymous missense *MC4R* variants identified in approximately 0.5 million people from the UK Biobank. The authors expressed these receptor variants in cultured HEK293 cells and studied their ability to stimulate G_s_-mediated cAMP accumulation, the recruitment of barr2, and the formation of pERK. Interestingly, GoF *MC4R* variants that showed a bias for barr2 recruitment were associated with a significantly lower risk of obesity and associated metabolic complications (9), suggesting that MC4R-dependent barr2 recruitment may play an important role in the regulation of body weight. This correlation was not seen with *MC4R* variants that preferentially stimulated G_s_-mediated cAMP accumulation (9). However, the authors observed a positive correlation between the degree of barr2 bias displayed by the different GoF *MC4R* variants and their ability to stimulate agonist-dependent ERK phosphorylation. Thus, the present study reveals the molecular and cellular underpinnings that can explain the observations by Lotta et al. (9). Approximately 6% of individuals with European ancestry carry barr2-biased GoF *MC4R* variants (9), indicative of the broad general translational relevance of our findings.

As mentioned earlier, the MC4R is able to stimulate G_s_ signaling and cAMP production with high efficacy. However, under certain experimental conditions, MC4R signaling can also stimulate G_q/11_ signaling in hypothalamic neurons (52, 53). Surprisingly, mice lacking Ga_s_ in PVN neurons showed normal feeding behavior, and MTII was able to suppress food intake in these mutant mice in the same fashion as in control mice (54), suggesting that G_s_ signaling is not essential for mediating the satiety-inducing effects of MC4R signaling. On the other hand, the Weinstein laboratory subsequently demonstrated that mutant mice lacking both Ga_q_ and Ga_11_ in the PVN display severe obesity and hyperphagia and fail to reduce food intake after MTII treatment (44), indicating that PVN Ga_q/11_ signaling is required for the proper regulation of food intake and body weight.

In 2020, the first MC4R agonist, setmelanotide, was approved by the FDA for the treatment of obesity resulting from various genetic disorders, including pro-opiomelanocortin, proprotein convertase subtilisin/kexin type 1 (PCSK1), and leptin receptor deficiency (16–18). Importantly, we show in the present study that the acute appetite-suppressing effect of setmelanotide requires the presence of barr2 in MC4R^+^ neurons (Figure 1K).

The outcome of the present study, together with the genetic and functional data published by Lotta et al. (9), suggests that the development of barr2-biased MC4R agonists may lead to clinically useful anti-obesity drugs endowed with increased efficacy and a more favorable side effect profile. Since MC4R-stimulated G_q/11_ signaling has been shown to be essential for the satiety-inducing effects of MC4R signaling, these agents should be designed to retain their ability to promote signaling via G_q_-type G proteins.

## Methods

### Sex as a biological variable.

Our study examined male mice. Because female mice with a C57BL/6 background gain little weight and do not develop glucose intolerance and insulin resistance when maintained on an HFD, we did not carry out any experiments with female mice.

### Drugs, reagents, commercial kits, and antibodies.

The sources of all drugs, reagents, antibodies, and mouse strains are listed in Supplemental Table 1.

### Generation of MC4R-specific barr2-KO mice and mouse maintenance.

To generate mice lacking barr2 specifically in MC4R-expressing cells, we used a breeding strategy involving homozygous floxed *barr2* mice (*barr2^fl/fl^*) (26) and *Mc4r-t2a-Cre* mice (The Jackson Laboratory, stock 030795) (short name: MC4R-Cre mice), which express Cre recombinase selectively in MC4R neurons (27). These mice were backcrossed for 6 generations to WT C57BL/6 mice. Heterozygous *barr2^fl/+^* mice carrying the *MC4R-Cre* transgene were then backcrossed to homozygous *barr2^fl/fl^* mice to produce *barr2^fl/fl^* *MC4R-Cre* mice (MC4R-barr2-KO mice), and *barr2^fl/fl^* littermates lacking the Cre transgene (control littermates). All mice were maintained on a C57BL/6 background, housed at room temperature (RT; 22°C), and fed a standard chow diet (Lab Diet, 5018; energy density 3.05 kcal/g). Animals were housed under a 12-hour light/12-hour dark cycle with ad libitum access to water and food. For a subset of experiments, male mice were switched to an HFD (D12492, Research Diets Inc.; 60% kcal from fat) and maintained on this diet for a minimum of 6–8 weeks before starting metabolic testing. Unless stated otherwise, male mice were used for metabolic studies.

### Body composition analysis.

The EchoMRI-100H Composition Analyzer (Echo Medical Systems) was used to measure the fat and lean mass in mice.

### Drug dosing and administration.

To measure MTII- and setmelanotide-induced anorectic effects, both drugs were dissolved in sterile saline and administered via i.p. injection after a 24-hour fast. For these studies, MTII was administered at a fixed dose of 200 μg per mouse, whereas setmelanotide was dosed based on body weight (2 mg/kg, i.p.) (55). For indirect calorimetry and immunostaining experiments (cFos and pERK), MTII was injected i.p. at a dose of 10 mg/kg. Control animals were treated with saline alone (100 μL, i.p.).

### In vivo metabolic tests.

In vivo metabolic tests were performed using standard procedures. Glucose tolerance tests (i.p.) were performed with mice that had been fasted overnight for approximately 16 hours. Blood glucose levels were measured before and after i.p. injection of a glucose bolus (1 or 2 g/kg, as indicated) at specific postinjection time points using a portable glucometer (Contour Glucometer, Bayer). For insulin tolerance tests, mice were fasted for 4 hours and then injected i.p. with human insulin (0.75 or 1 U/kg; Humulin, Eli Lilly). Blood glucose levels were measured in the same fashion as described for glucose tolerance tests. Plasma insulin and leptin levels were determined using commercially available ELISA kits according to the manufacturers’ instructions (Crystal Chem Inc. and R&D Systems, respectively).

### Food intake measurements.

To measure daily food intake, mice were single-housed and allowed to acclimate to this setting for 1 week. After this period, food intake was measured daily for at least 1 week. To study food intake after saline or drug treatment, mice were single-housed, fasted for 24 hours, and then injected i.p. with saline (100 μL), MTII (200 mg), or setmelanotide (2 mg/kg) 30 minutes before lights out at 6:00 pm. Food intake was then measured during the first 3.5 hours of the dark phase.

### AAV stereotaxic injections and surgery.

MC4R-Cre mice that were at least 7–8 weeks old were anesthetized with isoflurane and then placed into a stereotaxic apparatus (David Kopf Instruments, model 940A, with 923B mouse gas anesthesia head holder). The skull was exposed via a small incision, and a small hole was drilled (using a 0.45 mm drill bit) into the skull for virus injections. A Hamilton 10 μL syringe with a 30- or 33-gauge blunt-end needle was inserted into the brain for virus delivery. Postoperative analgesia was provided using Meloxicam-ER (ZooPharm; 2 mg/kg, s.c.). We performed bilateral stereotaxic injections into the PVN of MC4R-barr2-KO mice and control littermates (total injection volume: 200 nL per side) using the following viruses (AAV serotype 8): pAAV-EF1α-DIO-barr2-myc-P2A-Cerulean (VectorBuilder), AAV-DIO-mCherry (full name: AAV8-hSyn-DIO-mCherry; Addgene), or AAV-DIO-EGFP (Addgene). In a similar fashion, we carried out bilateral injections into the PVN using an AAV coding for a dominant-negative version of MEK1 (AAV8:FLEX-MEK1dn-P2A-mCherry) (46) or AAV-DIO-mCherry (control virus). The coordinates for all PVN injections were as follows (from bregma): anterior-posterior, –0.80 mm; lateral (from midline), ± 0.27 mm; dorsal-ventral, –4.90 mm. In a similar fashion, the AAV-DIO-mCherry virus was injected bilaterally into the PVN and DMH of MC4R-Cre mice for the FACS isolation of MC4R^+^ neurons. The coordinates for DMH injections were as follows (from bregma): anterior-posterior, –1.75 mm; lateral (from midline), ± 0.33 mm; dorsal-ventral, –5.15 mm (from skull surface). Mice were allowed to recover for at least 2 weeks after virus injections.

### IHC and imaging studies.

To monitor the expression of cFos and pERK in the PVN, mice were anesthetized with avertin and transcardially perfused with 4% paraformaldehyde (PFA) in 0.1 M phosphate buffer fixative (pH 7.4). The brain was then removed and stored for 1 day in 4% PFA at 4°C. On the following day, 30 μm thick slices of the hypothalamic region containing the PVN were prepared using a Leica vibratome VT1000S. The sections were washed with PBS and incubated overnight at 4°C with primary antibodies against cFos (1:1,000) or pERK (1:200) (both from Cell Signaling Technology) diluted in PBS and supplemented with 1% BSA and 0.1% Triton X-100 (Sigma-Aldrich 9036-19-5). Slices were then washed 3 times with PBS containing 0.1% Triton X-100 and incubated with fluorophore-conjugated secondary antibodies (Alexa Fluor 488, 1:1,000 dilution; A-11008,Thermo Fisher Scientific) for 2 hours at RT. Finally, slices were rinsed twice with wash buffer (PBS with 0.5% Triton X-100) and then mounted using Vectashield to preserve fluorescence (Vector Labs). Fluorescence images were taken with a Zeiss LSM 700 confocal microscope.

### FACS isolation of hypothalamic MC4R^+^ neurons.

Hypothalamic MC4R^+^ neurons were isolated from the PVN and DMH of MC4R-Cre mice after stereotactic injections of the AAV5-DIO-mCherry virus. Freshly dissected brain sections were dissociated into single-cell suspensions using the Neural Tissue Dissociation Kit-Postnatal Neurons (Miltenyi Biotec, 130-094-802) according to the manufacturer’s protocol, followed by filtration through a 70 μm MACS SmartStrainer and centrifugation at 300*g* for 10 minutes. For FACS, neuronal suspensions were blocked with 250 μL PBS supplemented with 5% FBS (Gibco) for 30 minutes at 4°C. Cells were then incubated with a Cy5-conjugated anti-MC4R antibody (Bioss, bs-11417R-Cy5) for 30 minutes at 4°C in the dark, washed with 2 mL PBS, and centrifuged at 350*g* for 5 minutes at 4°C. The pellet was resuspended in 250 μL PBS, and MC4R^+^ neurons expressing mCherry were isolated via FACS (BD Biosciences FACSAria II) for further studies.

### qRT-PCR studies with RNA prepared from FACS-sorted MC4R^+^ cells.

To measure *barr2* mRNA expression levels, RNA was extracted from TRIzol lysates prepared from FACS-sorted MC4R^+^ cells using the RNeasy mini kit combined with the RNase-free DNase set from QIAGEN, following the manufacturer’s protocol. cDNA was synthesized using SuperScript III First-Strand Synthesis SuperMix (Invitrogen). Real-time qPCR was performed using TaqMan technology (Applied Biosystems). Primers specific for the mouse *barr2* gene were obtained from Integrated DNA Technologies. RNA expression data were normalized relative to the expression of β-actin (*Actb*) (Integrated DNA Technologies).

The sequences were as follows: *Barr2*-forward 5′-AAGTCGAGCCCTAACTGCAA; *Barr2*-reverse 5′-GGTGAGGGTCACGAACACTT; *Barr2*-probe 5′-AGCGCGACTTTGTAGATCACCTGG; *Actb*-forward 5′-TTTCCAGCCTTCCTTCTTGG; *Actb*-reverse 5′-GGCATAGAGGTCTTTACGGATG; and *Actb*-probe 5′-TGGAATCCTGTGGCATCCATGAAACT.

### qRT-PCR studies with RNA prepared from PVN micro-punches.

MC4R-barr2-KO mice and control littermates were anesthetized with isoflurane and rapidly decapitated. Brains were removed quickly and sectioned on ice. The PVN was isolated using a 1.5 mm diameter micro-punch. PVN tissue punches were collected into bead-containing tubes and homogenized. Total RNA was extracted from PVN tissue using a TRIzol/phenol-chloroform extraction method following standard protocols. RNA concentration and purity were assessed, and 1–2 μg of total RNA was used for cDNA synthesis. cDNA was generated using the ZymoScript RT PreMix kit (Zymo Research) according to the manufacturer’s instructions. qRT-PCR studies were performed using SYBR Green Master Mix (Bio-Rad) under standard cycling conditions. Relative gene expression levels were normalized to β-actin as an internal control, and fold-changes in transcript levels were calculated using the ΔΔCt method. To determine Mc4r expression levels, the following primer pair was used (MilliporeSigma): *Mc4r*-F, 5′-ATCAATTCAGGGGGACACTG 3′ and *Mc4r*-R, 5′-GGCCATCAGGAACATGTGGA-3′.

### Immunoblotting.

Cell or tissue (PVN) lysates were homogenized in RIPA lysis buffer (Thermo Fisher Scientific) supplemented with protease and phosphatase inhibitors (Roche). Protein concentrations were determined by using the BCA method (Thermo Fisher Scientific). Equal amounts of protein (20 μg) were mixed with 4× SDS loading buffer and denatured at 75°C for 5 minutes. The denatured samples were then resolved using SDS-PAGE gradient gels and transferred onto a nitrocellulose membrane by semi-dry transfer (BioRad). The membrane was then blocked for 1 hour at RT using Tris-buffered saline with 0.1% Tween (TBS-T) containing 5% BSA. Subsequently, the membrane was incubated overnight at 4°C with primary antibodies (anti-tERK [9102, Cell Signaling Technology], anti–pERK-1/2 [9101, Cell Signaling Technology], anti-barr2 [Invitrogen, PA1-732], anti–histone 3 [ab1971, Abcam], or anti–β-actin [4970, Cell Signaling Technology]). For the detection of barr2 and pERK-1/2, blots (membranes) were cut into 2 parts. The upper part was probed either with an anti-barr2 antibody (Invitrogen, PA1-732) or an anti–pERK-1/2 antibody (Santa Cruz Biotechnology, clone E4). The lower part was used for detecting the loading control (histone 3 or β-actin). After overnight incubation with the primary antibody at 4°C, blots were washed extensively with TBS-T buffer and then incubated with the corresponding HRP-conjugated secondary antibody (anti-rabbit or anti-mouse at a dilution of 1:1,000) for 1 hour at RT. After intensive washing with TBS-T buffer, immunoreactive bands were detected by monitoring the ECL-dependent light emission with a chemiluminescence detection system (Peqlab or Azure Biosystems [PVN lysates]). The intensities of immunoreactive bands were quantified by densitometry using ImageJ (NIH).

### Indirect calorimetry studies.

TEE, RER, and locomotor activity (infrared beam break as total activity, 0.5″ spacing) were measured with an indirect calorimetry system (CLAMS using Oxymax software v5.52, Columbus Instruments). Mice were housed individually with ad libitum access to food (floor feeders) and water in chambers without bedding or nesting material (2.5 L volume, flow rate 0.5 L/min, sampling flow 0.4 L/min, settle time 55 seconds, measure time 5 seconds, each chamber sampled every 13 minutes). Locomotor activity was measured per 13-minute interval. All 12 calorimetry chambers were housed in a single temperature-controlled environmental chamber with a 12-hour light/12-hour dark cycle (lights on at 6 am). Mice were acclimated to the chambers for at least 2 days at 22°C, followed in order by continuous data recording of basal parameters at 22°C (24 hours), basal parameters at 30°C (24 hours), MTII (10 mg/kg, i.p.; single dose) or saline treatment at 30°C (24 hours), and a crossover treatment with saline or MTII (24 hours). The chamber temperature was changed at 6 am. MTII (single dose) was given at 10 am. The first 1.5 hours after MTII treatment were excluded from data analyses due to mast cell–mediated MTII-induced hypothermia (56).

### Cell culture.

The mouse hypothalamic cell line mHypoA-2/10 was purchased from Cedarlane (Clu-176) and cultured in DMEM supplemented with 10% FBS, 2 mM l-glutamine, and penicillin (100 U/mL and streptomycin (100 μg/mL).

### cAMP assay after barr2 knockdown.

In order to knock down barr2 expression, a predesigned *barr2* siRNA (Ambion, 4390771, ID: s103770) was introduced into mHypoA-2/10 cells by using lipofectamine RNAiMAX transfection reagent (Thermo Fisher Scientific,13778100). In parallel, cells were transfected with a scrambled control siRNA (Thermo Fisher Scientific, 4390846). For each cavity of a 6-well plate (Thermo Fisher Scientific,140675), 5 pmoles siRNA (10 μL) were mixed with 1.5 μL of lipofectamine in Opti-MEM (Thermo Fisher Scientific, 31985062). After 30 minutes at RT, 10 μL of this mixture was added to the bottom of the well, followed by the addition of approximately 400,000 cells in growth medium (2 mL). Then, 48 hours later, cells were labeled for 16 hours in serum-free DMEM containing 2.5 μCi/mL of [^3^H]adenine. Cells were then stimulated for 45 minutes at 37°C in DMEM containing 1 mM 3-isobutyl-1-methylxanthine and 100 nM MTII. The reaction was terminated by removing the medium and adding ice-cold 5% trichloroacetic acid to the cells. [^3^H]cAMP was then purified by sequential chromatography (use of Dowex-resin/aluminum oxide columns) (57), and the accumulation of [^3^H]cAMP was expressed as fmoles per 100,000 cells.

### ERK-1/2 activation assay after barr2 knockdown.

Using the same transfection strategy as described in the previous paragraph, mHypoA-2/10 cells were treated with either *barr2* siRNA or scrambled control siRNA. For each well of a 6-well plate (Thermo Fisher Scientific,140675), 5 pmol siRNA was mixed with 1.5 μL lipofectamine in Opti-MEM. After 30 minutes at RT, 10 μL of this mixture were added to the bottom of each well, followed by the addition of approximately 200,000 cells suspended in growth medium (2 mL). Next, 48 hours later, cells were serum-starved for 24 hours and then incubated in DMEM at 37°C for different periods of time in the presence or absence of MTII (100 nM). After this incubation step, cells were lysed by adding Laemmli buffer to the 6-well plates. Lysates were subjected to SDS-PAGE (10%) and Western blotting studies, as described under *Immunoblotting*. Immunoreactive proteins (barr2, pERK-1/2, and histone 3) were detected with specific primary antibodies (Supplemental Table 1) using a chemiluminescence detection system (Peqlab). The resulting signals were quantified by densitometry using ImageJ (NIH), and the ratios of the protein of interest and the loading control (histone 3) were calculated.

### Measurement of intracellular calcium levels.

To monitor changes in intracellular calcium levels, approximately 500,000 mHypoA-2/10 cells were seeded in 10 cm^2^ dishes and serum-starved the next day. After 24 hours, cells were labeled with 10 μM Fura-2-AM (Sigma-Aldrich, F088) in Hanks’ Balanced Salt Solution containing 10 mM HEPES and 0.02 % Pluronic F-127 (Sigma-Aldrich, P2443) for 30 minutes at 37°C. Cells were washed, removed from the dish with PBS containing 5 mM EDTA, and collected by centrifugation. Resulting pellets were resuspended in 800 μL HBBS containing 10 mM HEPES and seeded in white 96-well plates (90 μL per well). A FLUOstar Omega plate reader was used to detect emission at 510 ± 20 nm after excitation with 340 ± 20 nm or 380 ± 20 nm with a frequency of 0.72 seconds for 45 seconds. After 5 seconds, 10 μL of drug solution containing bradykinin (1 μM) or MTII (10 μM) were automatically injected.

### Statistics.

Data are presented as mean ± SEM for the indicated number of observations. Statistical significance was determined using 2-way ANOVA with the indicated post hoc tests or an unpaired Student’s *t* test (1 or 2 tailed), as appropriate. A *P* value of less than 0.05 was considered statistically significant. The specific statistical tests that were employed are indicated in the figure legends.

### Study approval.

All animal studies were performed according to the *Guide for the Care and Use of Laboratory Animals* (National Academies Press, 2011) and approved by the IACUC of the National Institute of Diabetes and Digestive and Kidney Diseases (NIDDK, NIH, Bethesda, Maryland, USA).

### Data availability.

Values for all data points found in graphs are in the Supporting Data Values file. All data needed to evaluate the conclusions in the paper are present in the paper and/or the supplemental materials.

## Author contributions

MR, LW, and JW conceived and designed the study. MR, LW, ZC, OG, HL, SZ, TG, and AB carried out, analyzed, and interpreted experiments. OG, TG, and JW supervised experiments. KK supplied important reagents. MR wrote the first draft of the manuscript. MR and JW jointly finalized the manuscript.

## Conflict of interest

The authors have declared that no conflict of interest exists.

## Funding support

This work is the result of NIH funding, in whole or in part, and is subject to the NIH Public Access Policy. Through acceptance of this federal funding, the NIH has been given a right to make the work publicly available in PubMed Central.

- NIH DK075021 (to MR, LW, and JW).

## Supplementary Material

Supplemental data

Unedited blot and gel images

Supporting data values

## Version 1 04/21/2026

In-Press Preview

## Version 2 06/08/2026

Electronic publication
