# Supplementary material for: Mice lacking β-arrestin-2 in melanocortin 4 receptor–expressing neurons show marked metabolic deficits
Source: JCI Insight. 2026 Apr 21;11(11):e202213. doi: 10.1172/jci.insight.202213 (PMC13313541; doi:10.1172/jci.insight.202213)
Supplement: Supplemental data [file jciinsight-11-202213-s084.pdf]

Supplemental Data

Mice lacking β-arrestin-2 in melanocortin 4 receptor-expressing neurons show marked metabolic deficits

Misbah Rashid, Lei Wang, Zhenzhong Cui, Oksana Gavrilova, Huiyan Lu, Kozo Kaibuchi, Sarah Zeitlmayr, Thomas Gudermann, Andreas Breit, Jürgen Wess

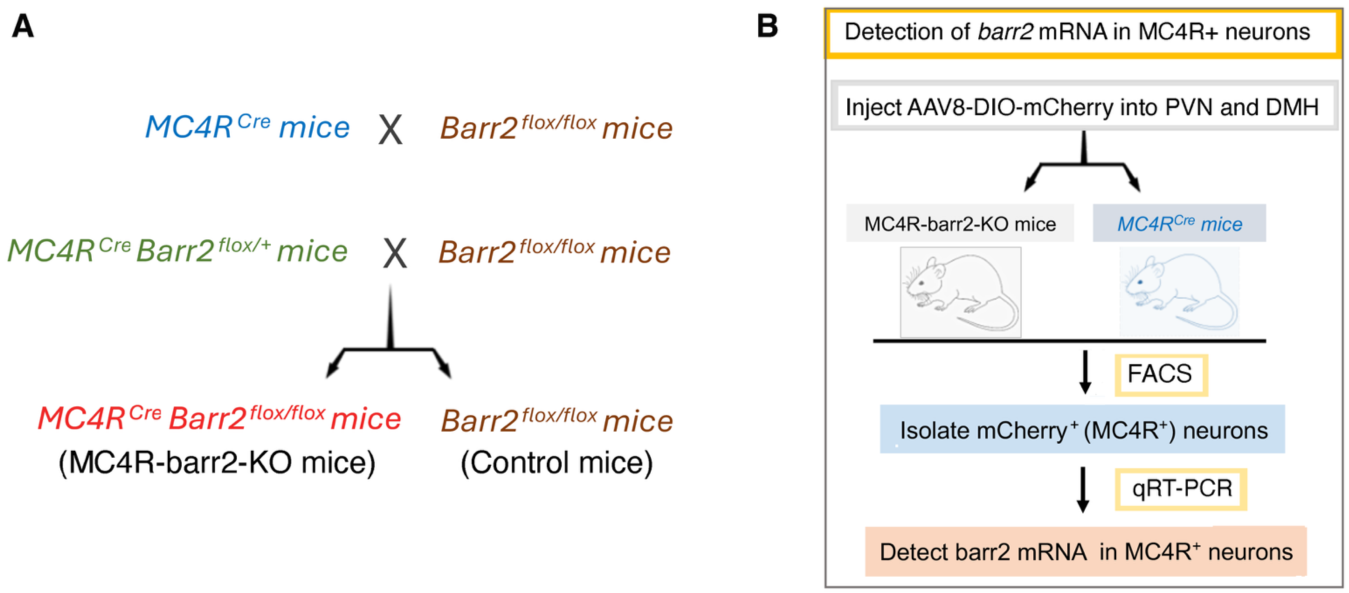

Supplemental Figure 1. Generation of MC4R-barr2-KO mice. (A) Breeding scheme used for generating mice lacking barr2 in MC4R-expressing cells. Heterozygous *MC4R-Cre* knockin mice were crossed with *barr2*$^{flox/flox}$ mice. *Barr2*$^{flox/+}$ mice carrying the *MC4R-Cre* transgene were then backcrossed to *barr2*$^{flox/flox}$ mice. This mating scheme yielded *barr2*$^{flox/flox}$ *MC4R-Cre* mice (MC4R-barr2-KO mice) and *barr2*$^{flox/flox}$ control littermates. **(B)** FACS strategy employed to detect the expression of *barr2* mRNA in MC4R-expressing hypothalamic neurons of the PVN and DMH. FACS, fluorescence-activated cell sorting; PVN, paraventricular nucleus; DMH, dorsomedial hypothalamus.

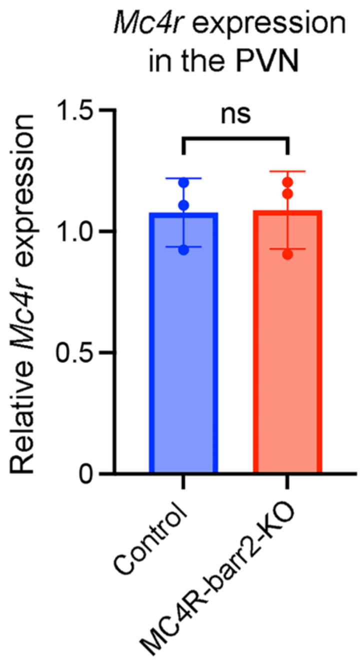

Supplemental Figure 2. *Mc4r* mRNA levels are similar in the PVN of MC4R-barr2-KO mice and control littermates. RNA was prepared from PVN punches obtained from MC4R-barr2-KO mice and control littermates (males). Subsequently, qRT-PCR studies were carried out to determine *Mc4r* expression levels. Relative gene expression levels were normalized using *β-actin* mRNA levels as an internal control. Data are given as means ± SEM (n = 3). ns, no statistically significant difference (two-tailed Student's t-test).

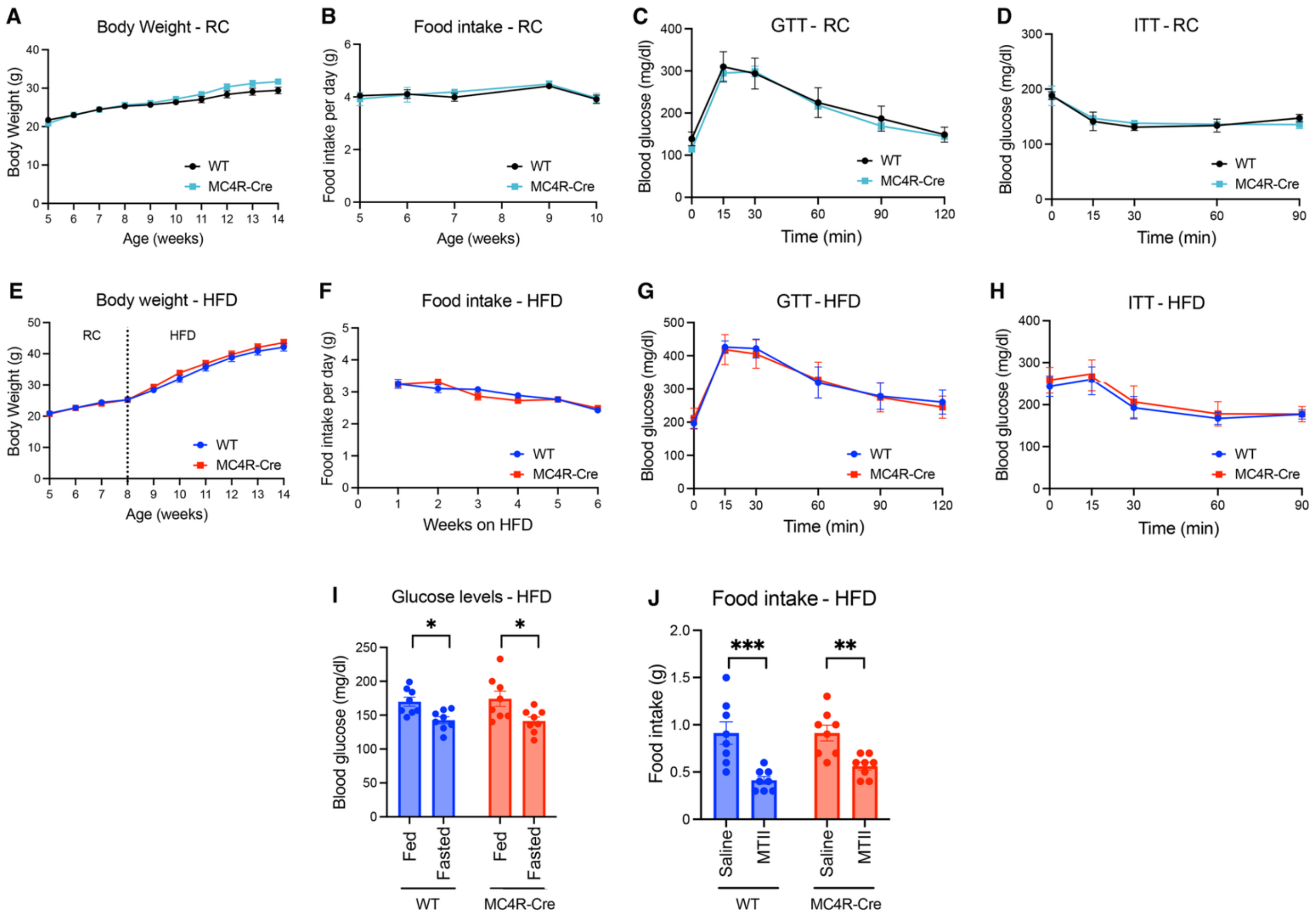

Supplemental Figure 3. The *MC4R-Cre* transgene does not cause any metabolic phenotypes on its own. MC4R-Cre mice (genetic background: C57BL/6) were crossed with WT C57BL/6 mice to generate MC4R-Cre mice and WT littermates. All studies were carried out with male mice. (**A–D**) Metabolic studies carried out with mice maintained on regular chow (RC). **(A)** Body weight gain. **(B)** Daily food intake measured over a 6-week period. **(C)** Glucose tolerance test (GTT). After an overnight fast, mice received an i.p. injection of glucose (2 g/kg). **(D)** Insulin tolerance (ITT). Following a 4-hr fast, mice were injected i.p. with insulin (0.75 U/kg). The data shown in **(B-D)** were obtained with 16-17-week-old mice. (**E–H**) Metabolic studies carried out with mice maintained on a high-fat diet (HFD). **(E)** Body weight gain. **(F)** Daily food intake measured over a 6-week period. **(G)** Glucose tolerance (GTT). After an overnight fast, mice received an i.p. injection of glucose (1 g/kg). **(H)** Insulin tolerance (ITT). Mice that had been fasted for 4-hr were injected with insulin (1 U/kg, i.p.). The data shown in (**F-H**) were

obtained with 14-17-week-old mice. **(I)** Fed and fasted blood glucose levels (mouse age: 19 weeks). **(J)** MTII-induced suppression of food intake. Single-housed mice that had been fasted for 24 hr were injected i.p. with either saline or MTII (200 μg) 30 min before lights out (6 pm). Food intake was measured during the first 3.5 hr of the dark phase (mouse age: 17–18 weeks). Data represent means ± SEM (n = 8 mice per group). *P < 0.05, **P < 0.01, ***P < 0.001 (two-way ANOVA followed by Šídák's multiple comparisons test).

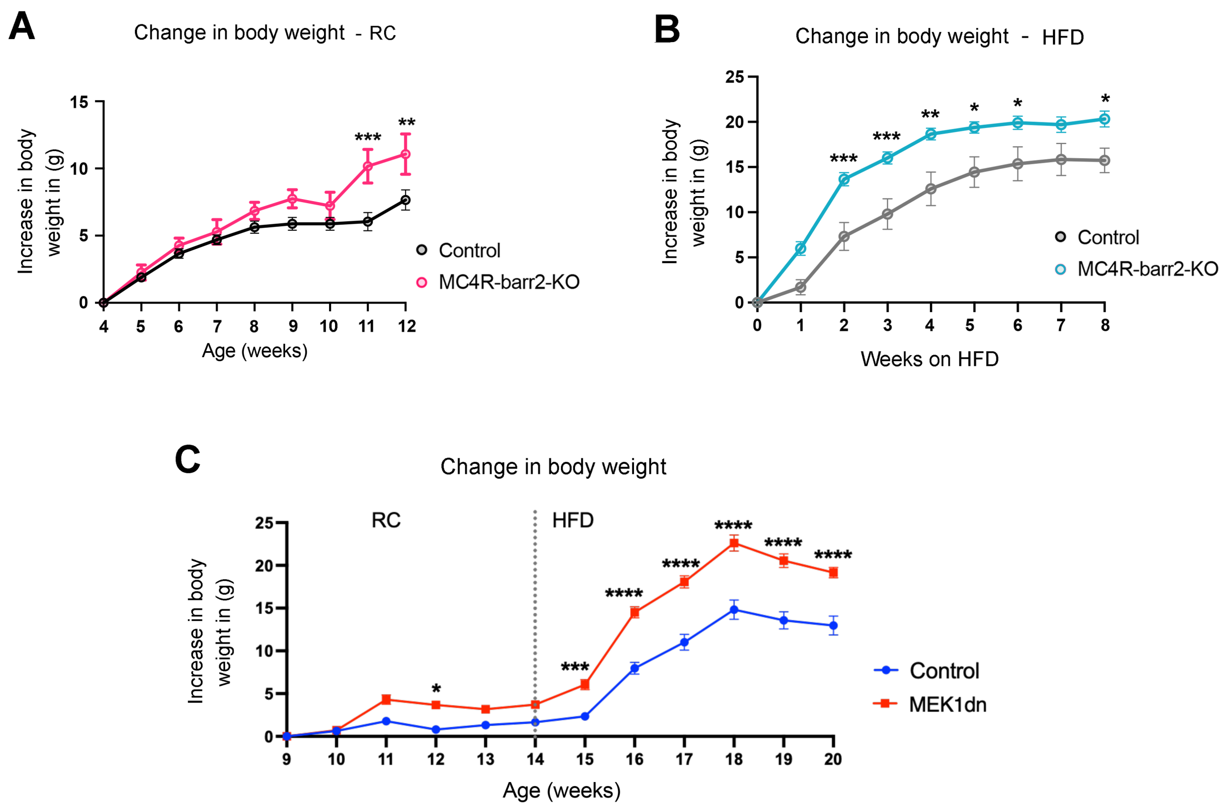

Supplemental Figure 4. Δ Body weight gain of control and mutant mice under different experimental conditions. All experiments were carried out with male mice. (**A**) Δ Body weight gain of MC4R-barr2-KO mice and control littermates maintained on regular chow (RC) (control, n = 9; MC4R-barr2-KO, n = 7). (**B)** Δ Body weight gain of MC4R-barr2-KO mice and control littermates maintained on a HFD (n = 8). Mice started to consume the HFD when they were 8-9 weeks old. (**C**) Δ Body weight gain of control and MEK1dn mutant mice consuming RC followed by HFD feeding (n = 7 or 8). Control, MC4R-Cre mice expressing mCherry in PVN MC4R+ neurons. MEK1dn, MC4R-Cre mice expressing MEK1dn in PVN MC4R+ neurons. Data are presented as mean ± SEM. *P < 0.05, **P < 0.01, ***P < 0.001, ****P < 0.0001 (two-way ANOVA with Šídák's multiple comparisons test). dn, dominant negative; MEK1, mitogen-activated protein kinase kinase 1.

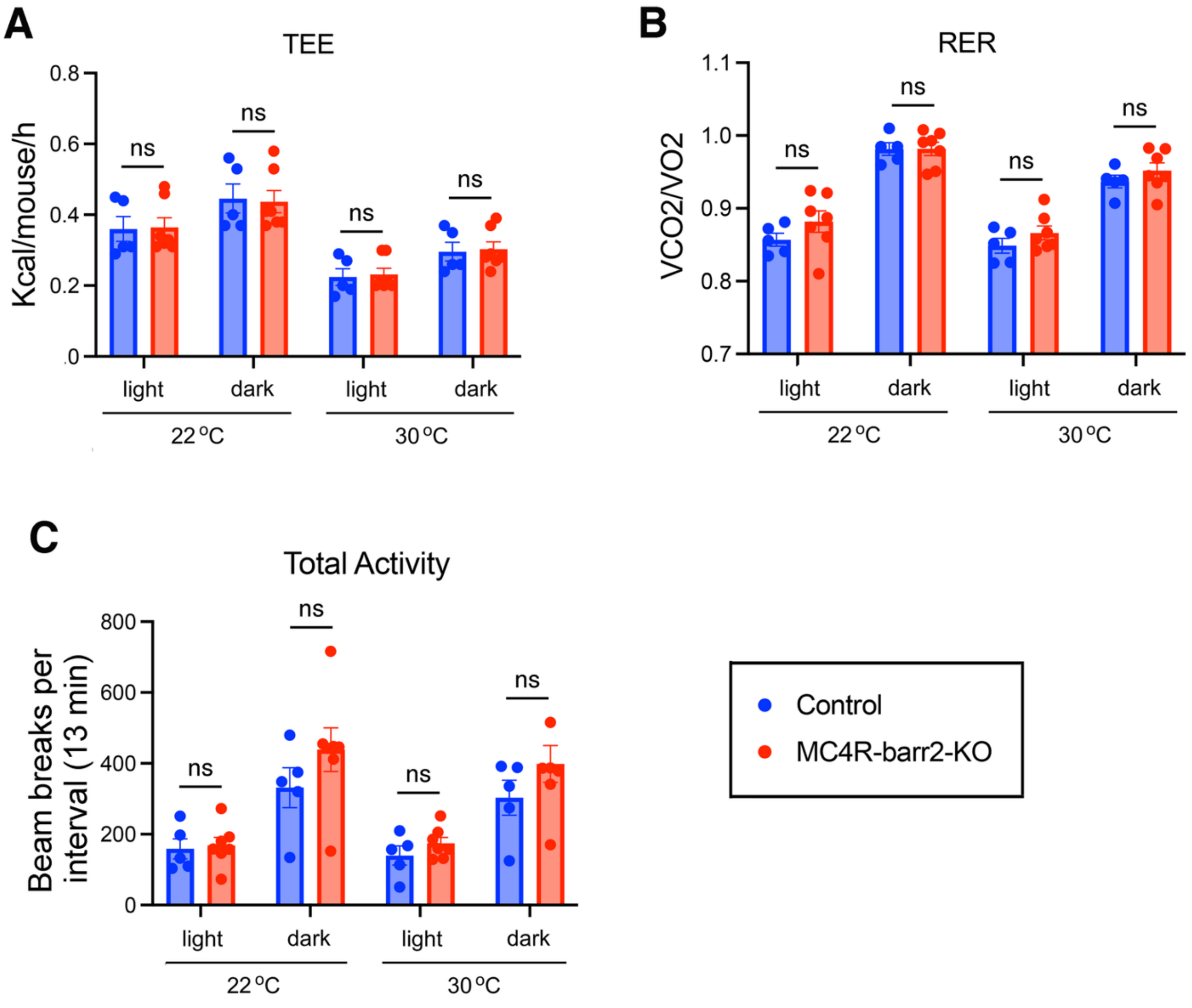

Supplemental Figure 5. Indirect calorimetry studies carried out with MC4R-barr2-KO and control mice consuming regular chow. MC4R-barr2-KO mice and control littermates (males; age: 12 weeks) maintained on regular chow were subjected to indirect calorimetry studies at room temperature (22 °C) and thermoneutrality (30 °C). **(A)** Total energy expenditure (TEE). **(B)** Respiratory exchange ratio (RER). **(C)** Total activity. Data show average values for the indicated 12-h light and dark periods presented as means ± SEM (control, n = 5; MC4R-barr2-KO, n = 7). ns, no statistically significant difference.

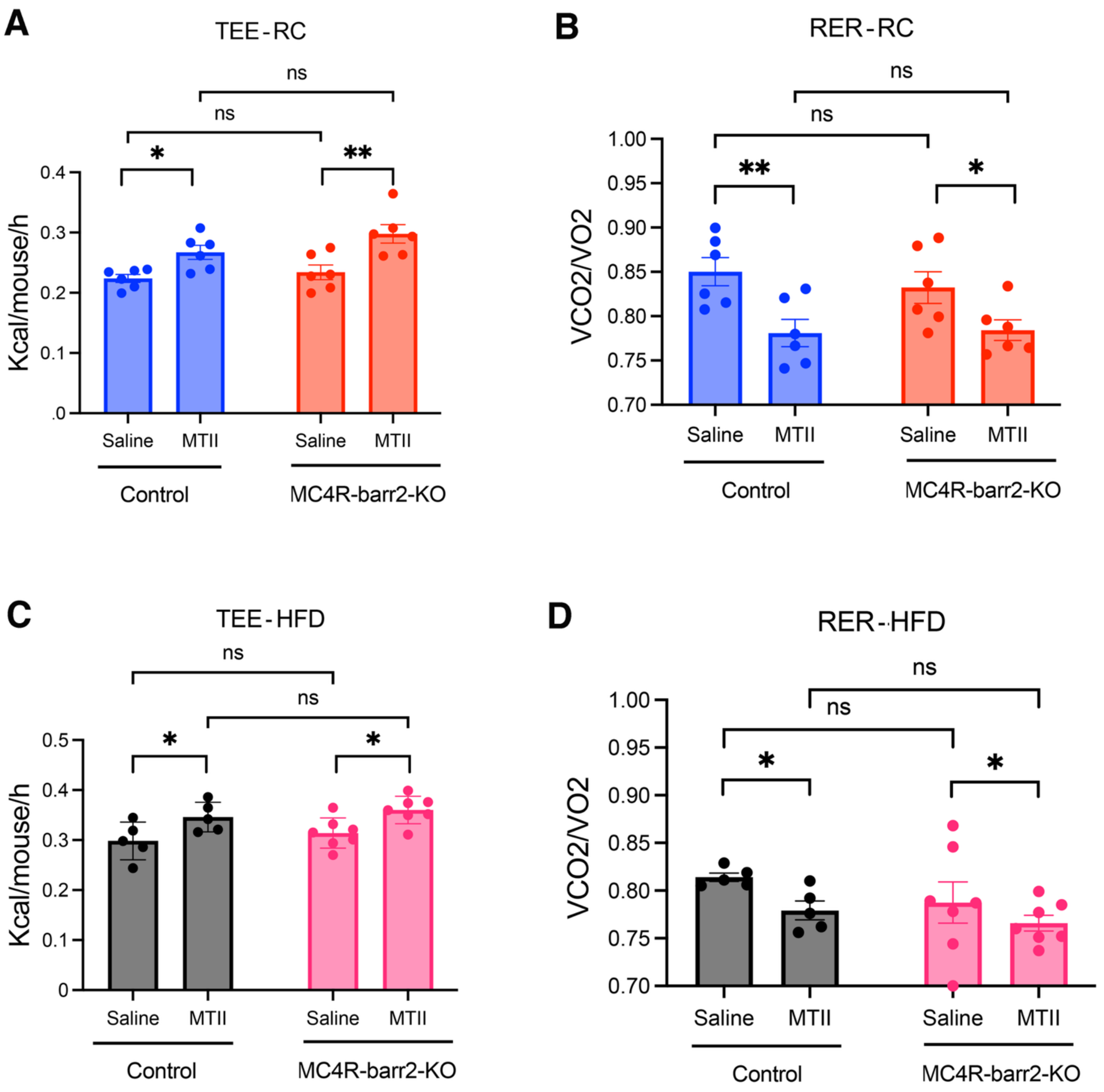

Supplemental Figure 6. Effects of MTII on energy expenditure and substrate utilization in control and MC4R-barr2-KO mice. MC4R-barr2-KO mice and their control littermates consuming regular chow (RC) or a high-fat diet (HFD) were subjected to indirect calorimetry studies at 30 °C. Mice were injected i.p. with a single dose of either saline or MTII (10 mg/kg), followed by measurements of total energy expenditure (TEE) and respiratory exchange ratio (RER). (**A, B**) Studies with RC mice (n = 6 per group; 12-week-old males). (**C, D**) Studies with HFD mice (control, n = 5; MC4R-barr2-KO, n = 7; 14-16-week-old males). Data are average parameters for the 1.5-3 hr post-injection period presented as means ± SEM. *P < 0.05, **P < 0.01 (two-way ANOVA followed by Šídák's multiple comparisons test). ns, no statistically significant difference.

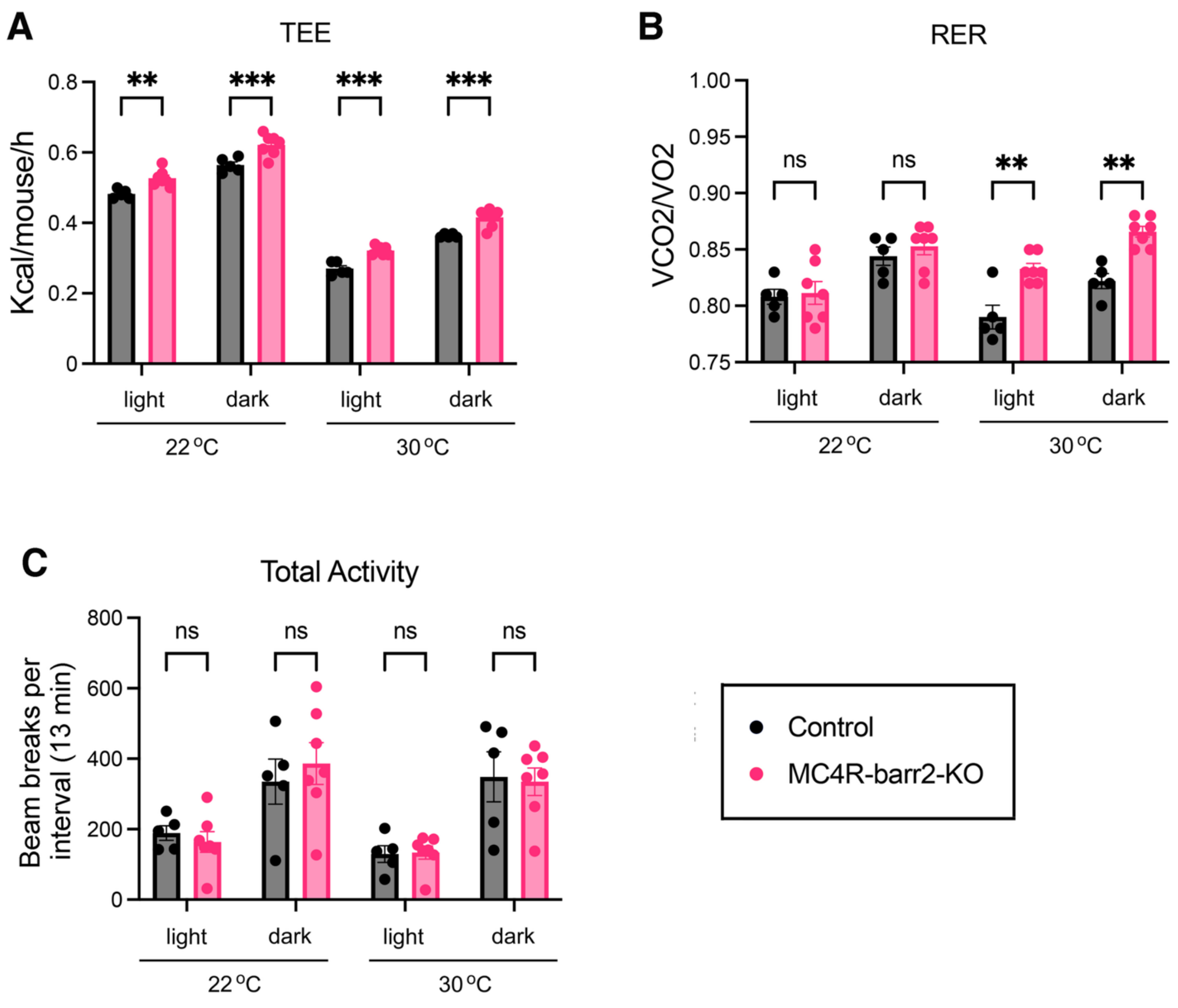

Supplemental Figure 7. Indirect calorimetry studies carried out with MC4R-barr2-KO and control mice maintained on a high-fat diet (HFD). MC4R-barr2-KO mice and control littermates (males; age: 13-14 weeks) maintained on a HFD for 10-12 days were subjected to indirect calorimetry studies at room temperature (22 °C) and thermoneutrality (30 °C). **(A)** Total energy expenditure (TEE). **(B)** Respiratory exchange ratio (RER). **(C)** Total activity. Data show average values for the indicated 12-h light and dark periods presented as means ± SEM. (control, n = 5; MC4R-barr2-KO, n = 7). $^{**}P < 0.01$, $^{***}P < 0.001$ (two-way ANOVA followed by Šídák's comparison tests). ns, no statistically significant difference.

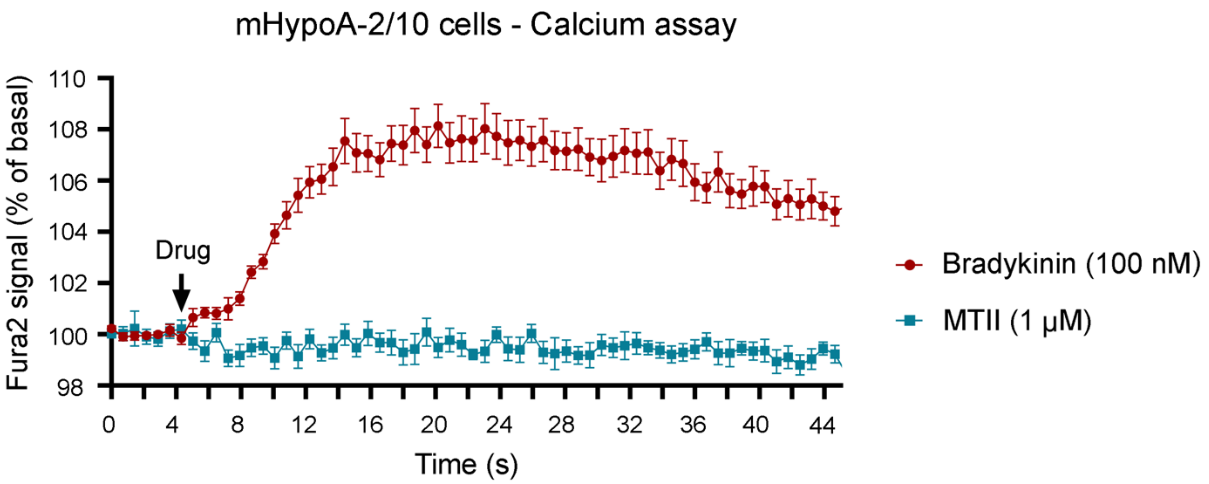

Supplemental Figure 8. MTII treatment of mHypoA-2/10 cells does not affect intracellular Ca^{2+} levels. Intracellular free Ca^{2+} concentrations ($[Ca^{2+}]_i$) were monitored by using a Fura-2 AM-based imaging technique (see Methods for details). Treatment of mHypoA-2/10 cells with MTII (1 µM) did not result in any detectable changes in $[Ca^{2+}]_i$. In contrast, bradykinin (100 nM), an agonist at G_q-coupled bradykinin receptors, caused a pronounced increase in $[Ca^{2+}]_i$. Data are given as means ± SEM (n = 3 independent experiments).

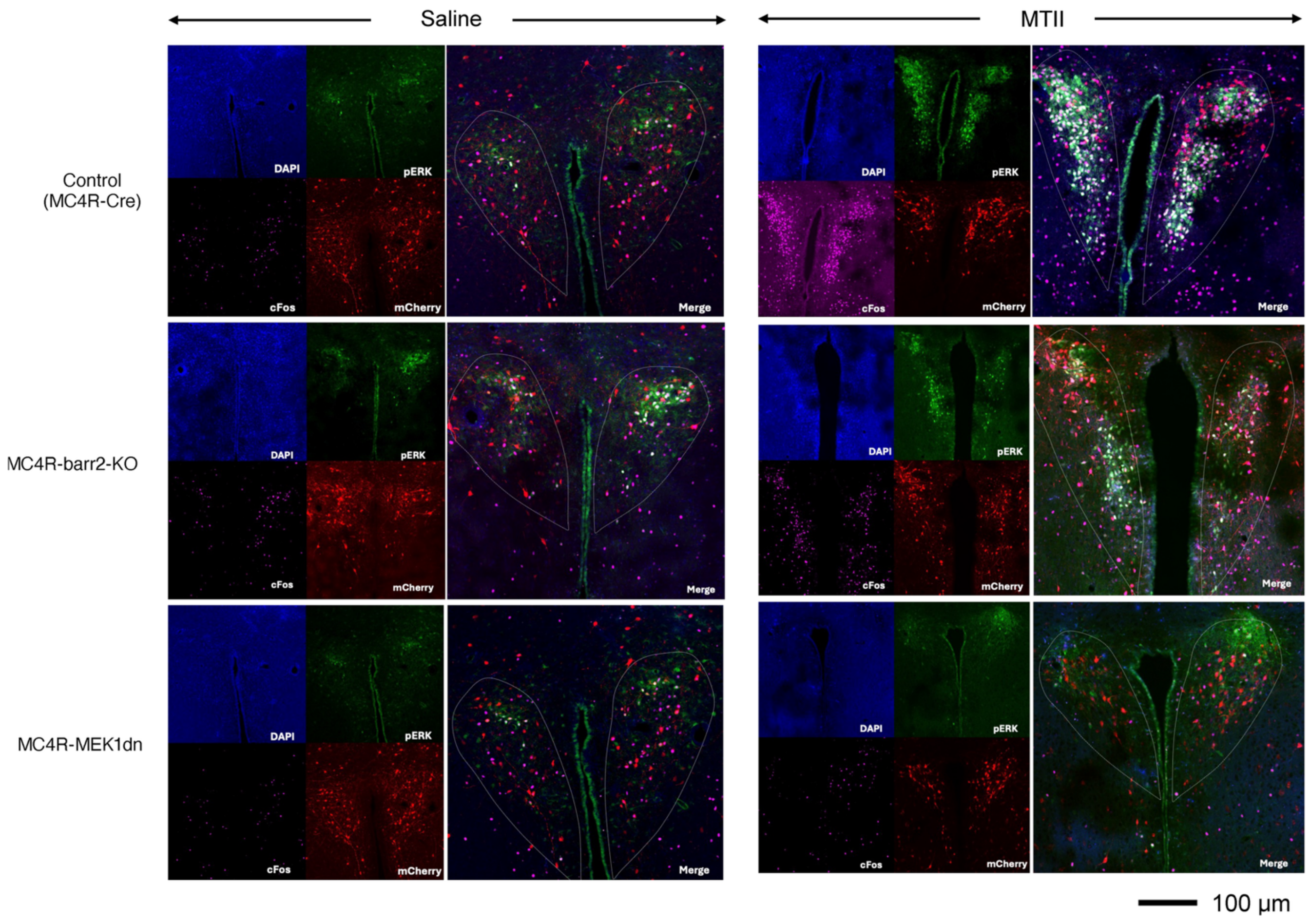

Supplemental Figure 9. Comparison of cFos and pERK signals in PVN neurons of control and MC4R mutant mice. Representative confocal images of hypothalamic brain sections containing the PVN from MC4R-Cre control mice, MC4R-barr2-KO mice (genotype: *barr2 flox/flox Mc4r-Cre*) and MC4R-MEK1dn mice (mice expressing MEK1dn in PVN MC4R+ neurons). Mice were injected with a single dose of saline or MTII, as described in Figures 6 and 7. MC4R-Cre control and MC4R-barr2-KO mice received the AAV-hSyn-DIO-mCherry control virus (bilateral injections into the PVN). MC4R-MEK1dn mice expressed mCherry in PVN MC4R+ neurons since these mice were treated with the AAV8::FLEX-MEK1dn-P2A-mCherry virus (bilateral injections into the PVN). Sections were immunostained for cFos (Alexa Fluor 555, magenta) and pERK (Alexa Fluor 488, green), and viral expression was visualized via mCherry fluorescence (red). Nuclei were counterstained with DAPI (blue). Dotted lines indicate PVN boundaries. Images were acquired using an Advanced Nikon N-SPARC confocal microscope.

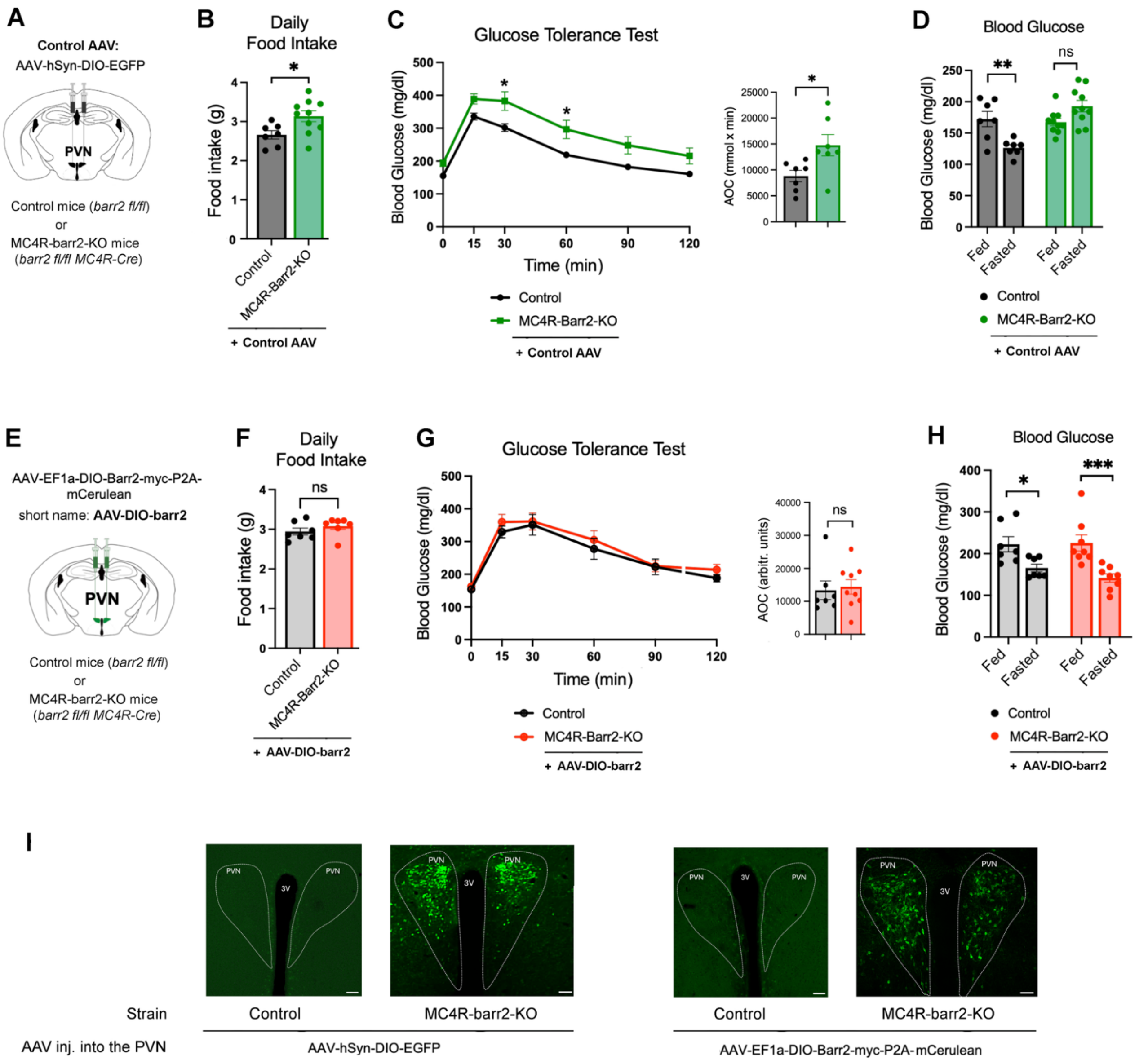

Supplemental Figure 10. AAV-mediated re-introduction of barr2 into the PVN of MC4R-barr2-KO mice restores normal food intake and glucose tolerance. All studies were carried out with male mice that had been maintained on a HFD for 16 weeks. (**A**) MC4R-barr2-KO mice (genotype: *barr2 flox/flox MC4R-Cre*) and control littermates (genotype: *barr2 flox/flox*) were injected bilaterally into the PVN with a control AAV (AAV-hSyn-DIO-EGFP). **(B–D)** Metabolic studies with mice injected with the control virus (control mice, n = 7; MC4R-barr2-KO, n = 10).

(B) Daily food intake of single-housed mice. **(C)** Glucose tolerance test. Mice received an i.p. bolus of glucose (1 g/kg) after an overnight fast for 16 hr. Bar graphs represent AOC values. **(D)** Blood glucose levels measured in the fed and fasted state (overnight fast for 16 hr). (**E**) MC4R-barr2-KO mice and control littermates were injected bilaterally into the PVN with AAV-EF1α-DIO-barr2-P2A-Cerulean (short name: AAV-DIO-barr2). **(F–H)** Metabolic studies with AAV-DIO-barr2-injected mice (control mice, n = 7; MC4R-barr2-KO mice, n = 8). **(F)** Daily food intake of single-housed mice. **(G)** Glucose tolerance test. Mice were treated with i.p. glucose (1 g/kg) after an overnight fast (16 hr). Bar graphs represent AOC values. **(H)** Blood glucose levels measured in the fed and fasted state (overnight fast for16 hr). (**I**) Representative immunofluorescence images indicating the selective targeting of AAV-hSyn-DIO-EGFP (left panel) and AAV-EF1α-DIO-barr2-P2A-Cerulean (right panel) to the PVN of MC4R-barr2-KO mice (see schemes (**A**) and (**E**)). Scale bars, 100 μm. Data are given as means ± SEM. Statistical significance was determined using two-way ANOVA followed by Šídák's multiple comparisons test, except for comparing AOC bars in panels (**C**) and (**G**) (two-tailed Student’s t-test). *P < 0.05; **P < 0.01; ***P < 0.001. AOC, area of the curve; ns, no statistically significant difference.

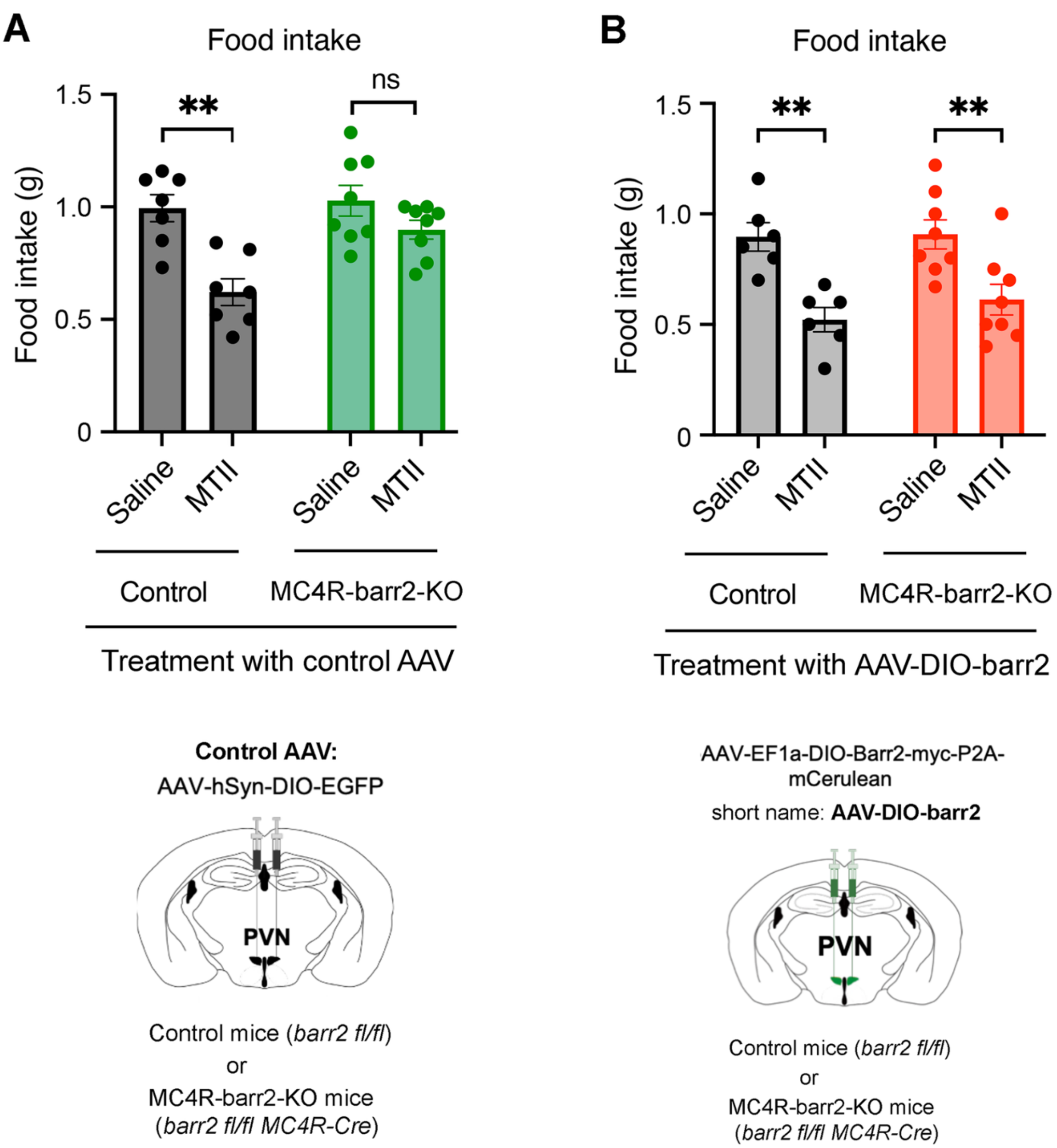

Supplemental Figure 11. MTII-induced suppression of food intake is restored in MC4R-barr2-KO mice after re-introduction of barr2 into the PVN. Food intake studies were carried out with male mice that had been maintained on a HFD for ~10 weeks. (**A, B**) Prior to MTII injections, MC4R-barr2-KO mice (genotype: *barr2 flox/flox MC4R-Cre*) and control littermates (genotype: *barr2 flox/flox*) were injected bilaterally into the PVN with either a control AAV (AAV-hSyn-DIO-EGFP) (**A**) or the AAV-EF1α-DIO-barr2-myc P2A-Cerulean virus (**B**) (control mice, n = 7; MC4R-barr2-KO mice, n = 8). After a 24-hr fast, single-housed mice were

injected i.p. with either vehicle (saline) or MTII (200 μg per mouse) 30 min before lights off. Food intake was then measured over the next 3.5 hr. Data are presented as means ± SEM. Statistical significance was determined using two-way ANOVA followed by Šídák's multiple comparisons test. $^{**}P < 0.01$. ns, no statistically significant difference.

Supplemental Table 1. Antibodies, drugs, reagents, kits, and mouse strains used in this study

Reagents	Source	Cat. # (identifier)
**Antibodies**		
Alexa Fluor 488	Thermo Fisher Scientific	A-11008
Anti-β-actin mouse mAb	Santa Cruz	SC-47778
Anti-cFos Ab	Cell Signaling	2250S
Anti-ERK-1/2 Rb Ab	Cell Signaling	9102
Anti-p-ERK-1/2 Rb Ab	Cell Signaling	9101
HRP-linked secondary Ab (Rb)	Cell Signaling	7074
HRP-linked secondary Ab (mouse)	Cell Signaling	7076
Anti-pERK Ab	Santa Cruz	Clone E4
Anti-Barr2 Ab	Invitrogen	PA1-732
Anti-Histone-3 Ab	Abcam	ab1791
Cy5-conjugated anti-MC4R Ab	Bioss	bs-11417R-Cy5
**Chemicals, reagents, peptides, reagents**		
Agarose	Thermo Fisher Scientific	16500-500
Emerald AMP GT PCR Master Mix	Takara	RR310
Triton X-100	Sigma-Aldrich	9036-19-5
PBS	KD Medical	RGF-3190
Isoflurane	Baxter Healthcare Corporation	10019-360-40
Avertin (2,2,2-Tribromoethanol)	Sigma	T48402
Meloxicam-ER 2mg/ml	ZooPharm	N/A
DMEM - high glucose	Sigma-Aldrich	D5796
Fetal bovine serum	Sigma-Aldrich	F4135
Opti-MEM	Thermo Fisher Scientific	31985062
Lipofectamine RNAimax	Thermo Fisher Scientific	13778100
RIPA lysis and extraction buffer	Thermo Fisher Scientific	89900
Bovine serum albumin	GoldBio	A-420-250
Protease Inhibitor Cocktail	Sigma-Aldrich	11836170001
TRIzol	Invitrogen	15596026
Tween 20	Sigma-Aldrich	P7949
10x TBS	KD Medical	RGF-3385
Trypsin-EDTA solution	Sigma-Aldrich	T4049
NuPAGE 4-12% Bis-Tris protein gel	Invitrogen	NP0336BOX
NuPAGE MOPS SDS running buffer (20x)	Invitrogen	NP0001

NuPAGE 3-8% Tris-Acetate protein gel	Invitrogen	EA03785BOX
Nitrocellulose transfer packs	BioRad	1704158
Nitrocellulose membranes	Amersham Protran™ 0.45 µm	10600002
NuPAGE LDS sample buffer (4X)	Invitrogen	NP0007
SuperSignal West Dura Extended Duration Substrate	Thermo Fisher Scientific	34076
SuperFemto ECL Chemiluminescence Kit	Vazyme	E423-02
Vectashield	VectorLabs	H-1000-10
IBMX	Sigma-Aldrich	I5879
[^{3}H]Adenine	PerkinElmer	NET063005MC
Trichloroacetic acid	Sigma-Aldrich	T6399
Laemmli buffer	BioRad	1610737
Pluronic F-127	Sigma-Aldrich	P2443
Barr2 siRNA	Ambion	4390771-s103770
Control siRNA	Thermo Fisher	4390846
Insulin (Humulin R)	Eli Lilly	00002821501
Melanotan II (MTII)	Fisher Scientific	50259784
Setmelanotide	MedChemExpress	HY-19870
Bradykinin	Sigma-Aldrich	B3259
Fura-2-AM	Sigma-Aldrich	F088
**Commercial assays/kits**		
BCA protein assay kit	Thermo Scientific	23225
Ultra-Sensitive Mouse Insulin ELISA Kit	Crystal Chem	90082
Leptin Quantikine ELISA Kit	R&D Systems	MOB00B
ZymoScript RT PreMix Kit	Zymo Research	R3012-1-1
Neural Tissue Dissociation Kit	Miltenyi Biotec	130-094-802
**Cell lines**		
mHypoA-2/10	Cedarlane	CLU176
**Experimental models: Animals**		
Barr2 flox/flox mice	Dr. Marc Caron, Duke University, Durham, NC	N/A
MC4R-barr2-KO mice	Generated at NIDDK	N/A
MC4R-Cre mice	The Jackson Laboratory	030795
Wildtype C57BL/6 mice	Taconic	B6NTac

**Recombinant viruses**		
AAV8::FLEX-MEK1dn-P2A-mCherry	Vector Biolabs	N/A
AAV-hSyn-DIO.mCherry	Addgene	50459-AAV8
AAV-hSyn-DIO-EGFP	Addgene	50457-AAV8
AAV-EF1α-DIO-barr2-P2A-Cerulean	provided by Dr. Nikhil Urs, University of Florida, Gainesville, FL	N/A
