# Supplementary material for: Mice lacking β-arrestin-2 in melanocortin 4 receptor–expressing neurons show marked metabolic deficits
Source: JCI Insight. 2026 Apr 21;11(11):e202213. doi: 10.1172/jci.insight.202213 (PMC13313541; doi:10.1172/jci.insight.202213)
Supplement: Unedited blot and gel images [file jciinsight-11-202213-s085.pdf]

anti-beta-arrestin-2

66kDa

anti-histone-3

25kDa

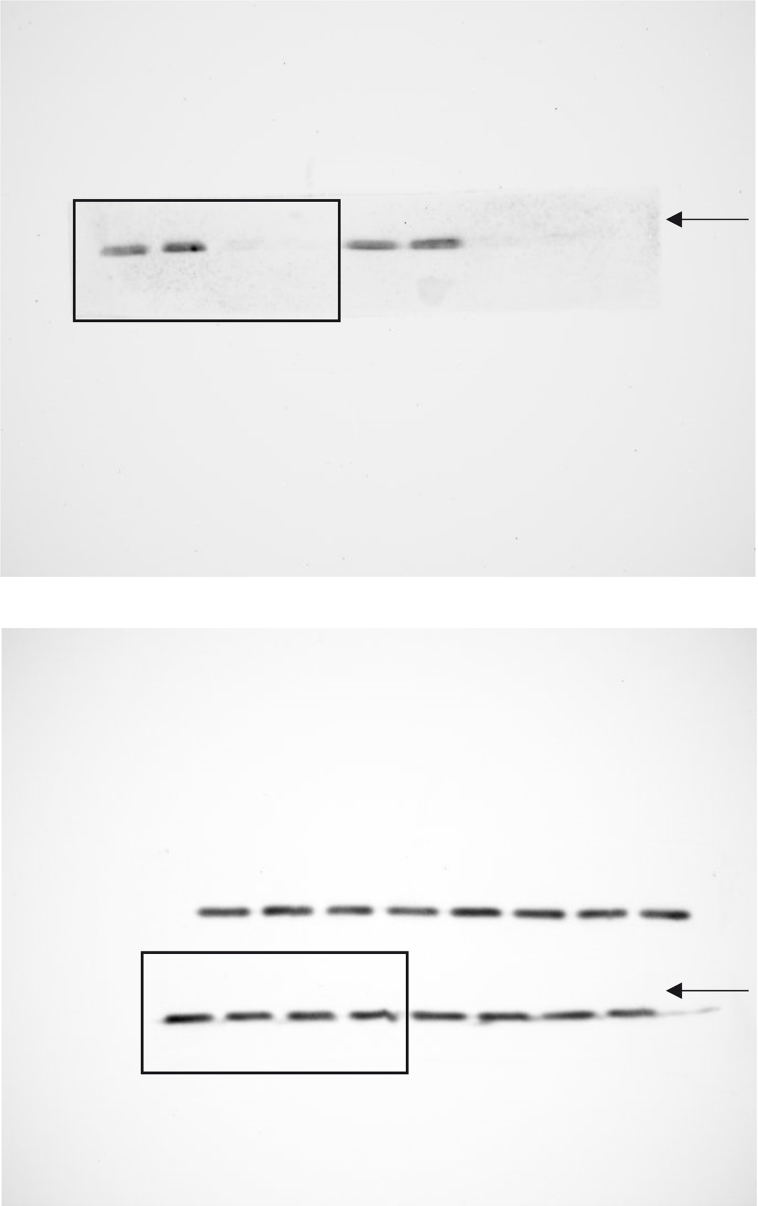

Unedited blots for Fig. 3A

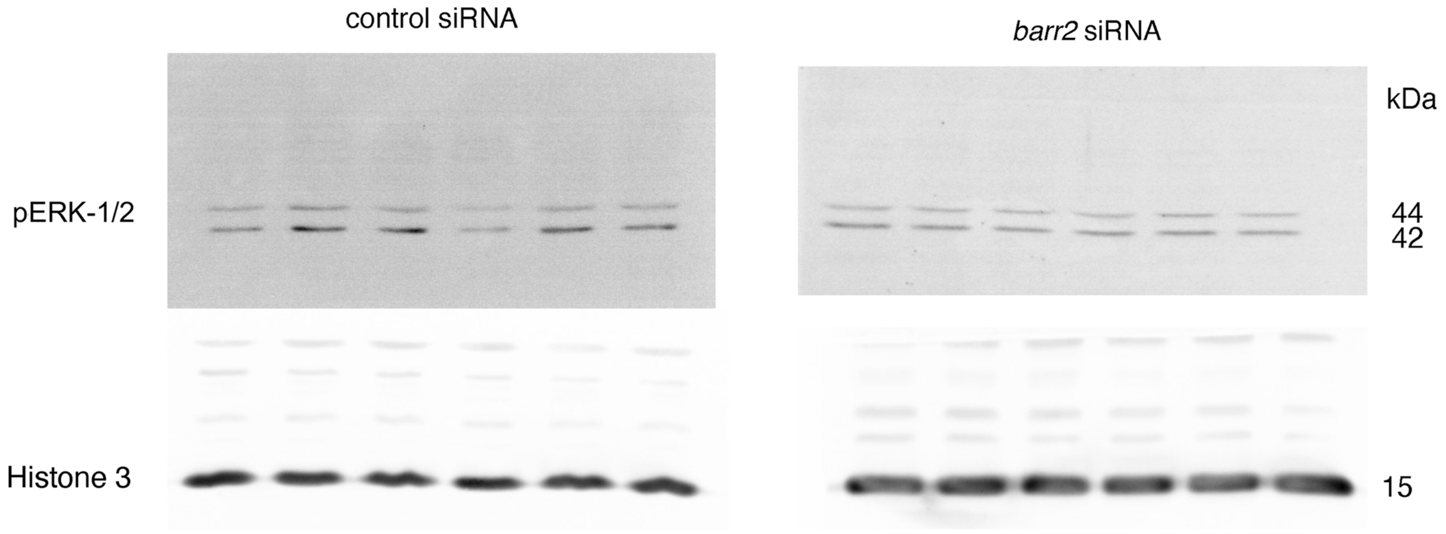

Unedited blots for Fig. 3C

Total ERK

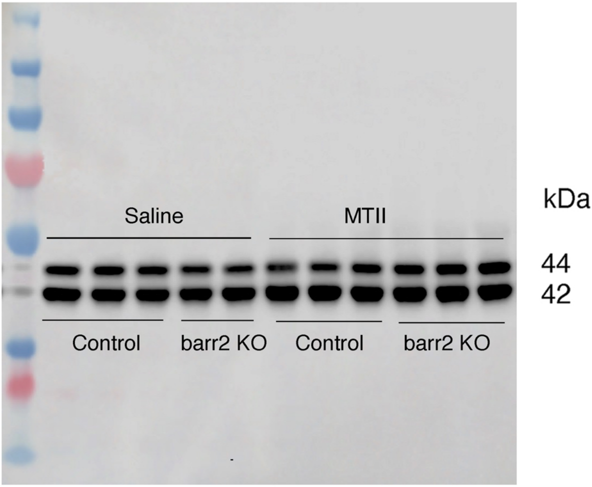

pERK

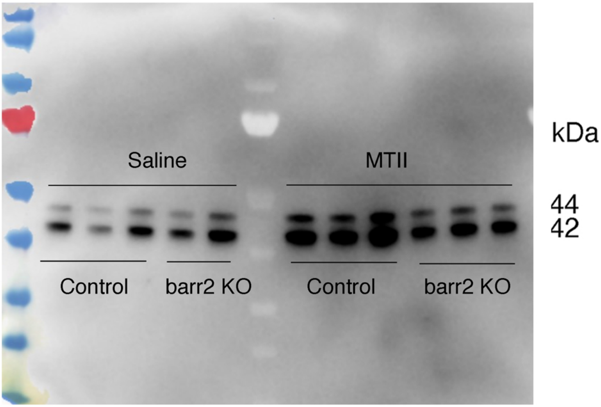

Full unedited gels
for Fig. 3E
